# Postovulatory maternal transcriptome in Atlantic salmon and its relation to developmental potential of embryos

**DOI:** 10.1186/s12864-019-5667-4

**Published:** 2019-04-24

**Authors:** Teshome Tilahun Bizuayehu, Maren Mommens, Arvind Y. M. Sundaram, Anusha K. S. Dhanasiri, Igor Babiak

**Affiliations:** 1 Faculty of Biosciences and Aquaculture, Nord University, N-8049 Bodø, Norway; 2 Present address: Sars Center, University of Bergen, N-5006 Bergen, Norway; 3 Aqua Gen AS, P.O.Box 1240, Sluppen, N-7462 Trondheim, Norway; 4 Department of Medical Genetics, Oslo University Hospital and University of Oslo, P. O. Box 4956, Nydalen, 0424 Oslo, Norway

**Keywords:** Atlantic salmon, Egg quality, Hepcidin-1, Maternal factors, miRNA, mRNA, Postovulatory aging, RNA-seq

## Abstract

**Background:**

Early development of an oviparous organism is based on maternally stocked structural, nutritional and regulatory components. These components influence the future developmental potential of an embryo, which is referred to as egg quality. Until zygotic genome activation, translational activity in a fish early embryo is limited to parentally inherited transcripts only. In this study, we asked whether egg transcriptome is associated with egg quality in Atlantic salmon (*Salmo salar*), which is capable of storing ovulated eggs in its abdominal cavity for a long time before spawning.

**Results:**

We analyzed messenger RNA (mRNA) and micro RNA (miRNA) transcriptomes throughout the post-ovulatory egg retention period in batches of eggs from two quality groups, good and poor, classified based on the future developmental performance. We identified 28,551 protein-coding genes and 125 microRNA families, with 200 mRNAs and 5 miRNAs showing differential abundance between egg quality groups and/or among postovulatory ages. Transcriptome dynamics during the egg retention period was different in the two egg quality groups. We identified only a single gene, *hepcidin-1*, as a potential marker for Atlantic salmon egg quality evaluation.

**Conclusion:**

The overlapping effect of post-ovulatory age on intrinsic egg developmental competence makes the quantification of egg quality difficult when based on transcripts abundance only.

**Electronic supplementary material:**

The online version of this article (10.1186/s12864-019-5667-4) contains supplementary material, which is available to authorized users.

## Background

Egg quality in fish is determined by multiple genetic and environmental factors acting together, and their effects are often difficult to predict due to the limited understanding of the underpinning mechanisms [1]. New approaches are needed to better understand the biological processes resulting in further developmental competence of eggs. In addition to inherited DNA epigenetic features [2, 3], mature oocytes contain components essential for further embryonic development. These essential nutrients, maternal RNAs and proteins are acquired to oocytes in the course of maturation process [4]. The acquired molecules are not only stored passively but also have functions throughout the oogenesis [5]. Therefore, the final set of oocyte constituents is resulting from both oogenesis progression history and maternal-to-embryo depository [4, 6]. Both groups of components can affect the future developmental potential of an embryo.

Majority of ray-finned fish species ovulate mature oocytes into an ovarian lumen or directly into abdominal cavity, where they are stored until spawning. This postovulatory retention period varies in teleosts from hours to weeks, depending on species, and the deterioration in oocyte developmental competence progresses in time accordingly [7]. In salmonids, postovulatory retention of ova in the coeloma can last for several weeks, depending on temperature [8], and the quality of eggs declines with an increase in postovulatory age [9]. In Atlantic salmon (*Salmo salar*), postovulatory aging shows the effect on egg developmental competence after 2-week retention [10].

Although the physical appearance of eggs, buoyancy, or ovarian fluid pH measurements provide some clues about the quality of eggs, still the fertilization success and subsequent normal embryonic development remain the most reliable measures of egg quality [11]. However, a posteriori quality check is not the ideal measure for the aquaculture industry, as certain resources are allocated to the production process prior to the measure.

Genetic marker-assisted screening of good egg-bearers helps to sort out the genetic background of lowered quality of eggs; however, DNA markers will not help to determine postovulatory age-related features occurring in an egg batch. Under conditions of commercial production, it is not possible to determine precisely the postovulatory age of every batch of eggs. Previous studies on fish egg transcriptome (reviewed by [12]) have suggested the potential of transcriptomics for evaluating embryonic developmental competence. Maternal transcripts are essential during the early embryonic development and for zygotic genome activation [13, 14]. The level of maternally stocked transcripts, such as *nucleoplasmin*, *tubulin β*, *insulin-like growth factor 1*, *keratin 8*, or *prohibitin 2*, are linked to the egg quality in rainbow trout (*Oncorhynchus mykiss*) [9, 15]. Maternally inherited *nucleoplasmin* is suggested as essential for zebrafish egg developmental competence [14].

An ovum is considered quiescent regarding translational activities. Maternal mRNAs in ova are non-polyadenylated and bound by proteins that prevent their translation before the oocyte activation [16]. Maternal mRNAs are activated by polyadenylation at different time and pace [17]. Timely degradation of maternal transcripts is important for embryonic development [16]. Micro RNAs (miRNAs) augment the mRNA degradation. miRNAs are ~ 22 nucleotides-long non-coding RNAs that serve as templates for RNA-protein assembly to degrade or suppress target transcripts through complementary binding. Although different types of miRNAs are found in fish oocytes [18], their role after oocyte maturation has not been elucidated yet.

In this study, we analyzed transcriptome repertoires in Atlantic salmon eggs during the postovulatory retention period in order to determine whether postovulatory aging affects the transcriptome dynamics and if yes, how such the effect is related to the egg quality determined a posteriori by developmental potential. We characterized maternal mRNA and miRNA transcriptomes and analyzed differential abundance of transcripts in relation to egg quality and postovulatory age.

## Results

### Sequencing, transcript identification and annotation

The sequencing resulted in 640,276,968 of 75 nts single reads. In total, 136,077 different transcripts were identified. Among them, after excluding low-count reads (< 1 RPKM) and counting at gene level, 28,551 genes were annotated as conserved mRNAs, which is 48.4% of non-repeat associated loci of salmon genome. The total number of sequences and annotation statistics for each sample is depicted in Additional file 1. The rest of the sequences could be unannotated transcripts and/or potential novel genes.

**Fig. 1:**
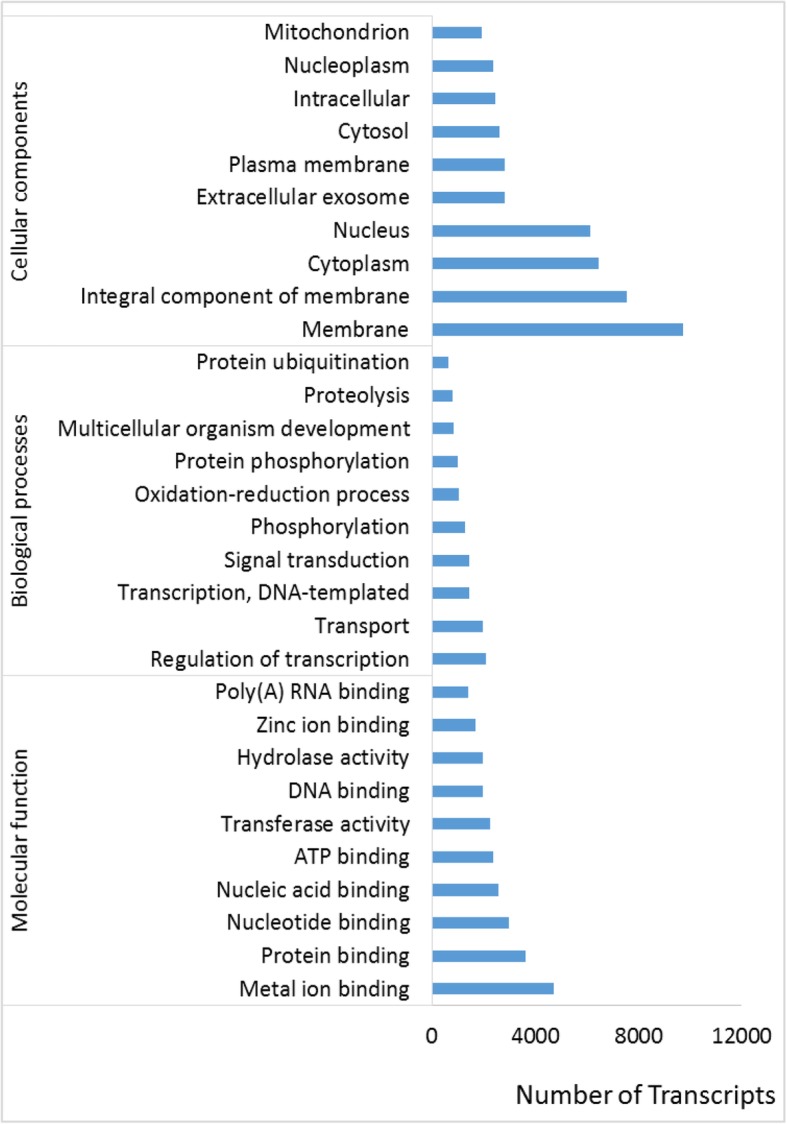
Gene Ontology (GO) classification for transcriptome from Atlantic salmon eggs. Only the top-10 significantly enriched GO-terms are presented for the three categories: biological processes, cellular components, and molecular functions. X-axis shows the number of unique genes enriched in the displayed terms. The detailed GO annotation is given in Additional file 2

In total, 23,636 transcripts were assigned to 14,197 GO-term IDs (Additional file 2). They were classified under categories: biological process, molecular function, and cellular components (Fig. 1, Additional file 2).

### Differential abundance of mRNA

**Fig. 2:**
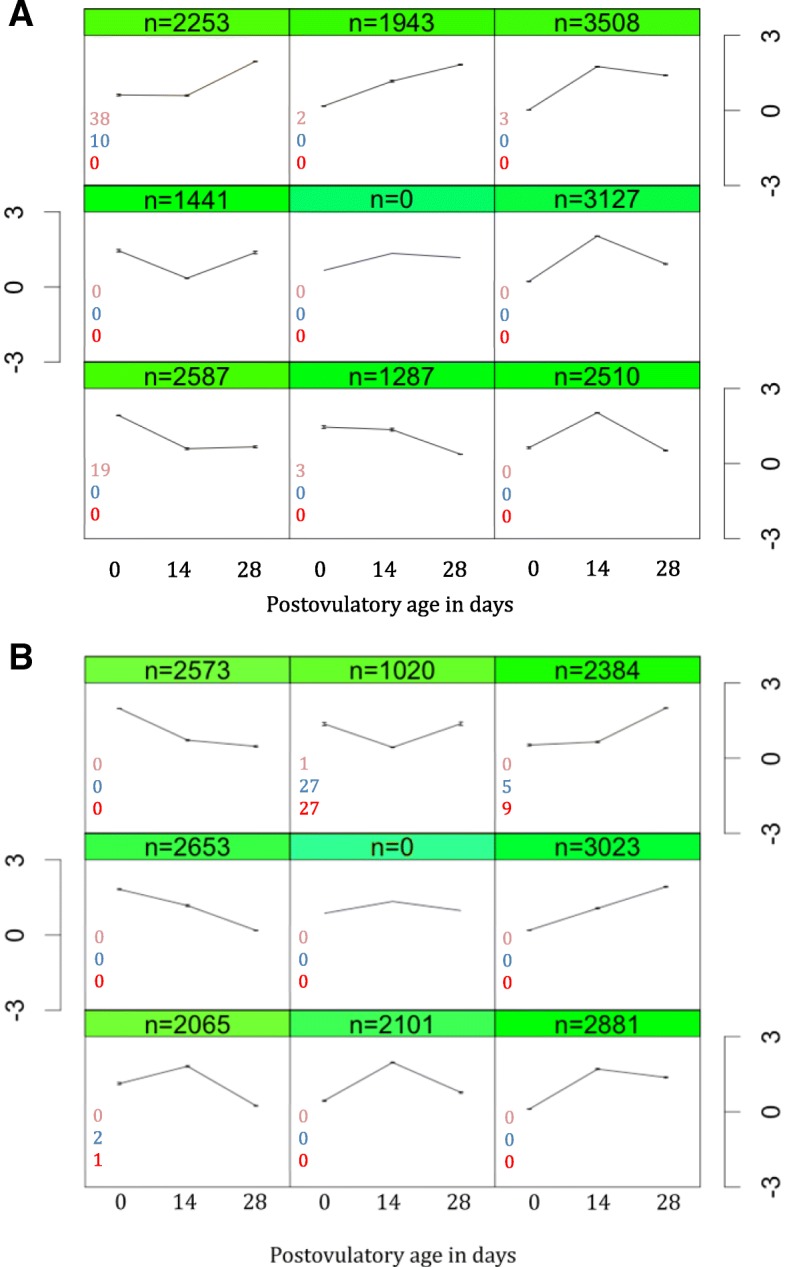
Dynamics of postovulatory transcriptome in Atlantic salmon egg obtained from **a**) good and **b**) poor egg quality group. Transcripts with similar expression pattern were clustered and represented by the scaled mean expression value at each sampling time. The dynamics of transcripts was obtained by self-organizing map clustering of individual transcripts at three time points: at the onset of ovulation, at 14, and at the 28 days post-ovulation (0dpo, 14dpo, and 28dpo, respectively). Number of differentially accumulated transcripts between the sampling time-points is shown for 28dpo vs 0dpo (orange), 28dpo vs 14dpo (blue) and 14dpo vs 0dpo (red) comparisons. The details of discrimination between the two quality groups are given in the text (see also Additional file 3). Y-axis is scaled expression level of transcripts. n is the number of transcripts showing a given expression pattern during the postovulatory aging. Nine different patterns for each egg quality group are displayed

**Fig. 3:**
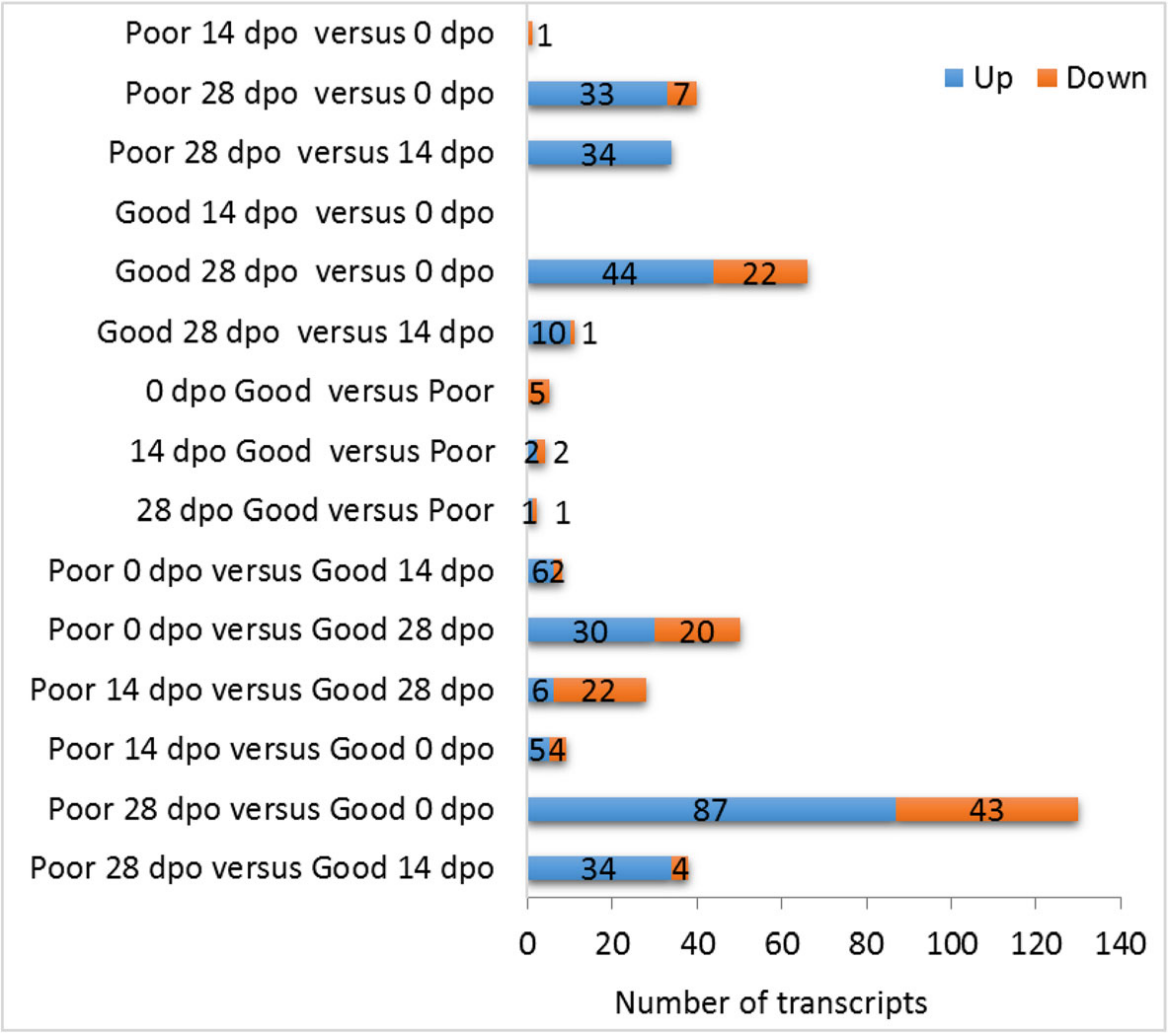
Number of significantly enriched transcripts in Atlantic salmon eggs throughout the postovulatory retention in the body cavity. Comparison of good versus poor quality egg batches (*n* = 6 each) in the three time points (days post-ovulation, dpo) and comparison of the time points within each egg quality group are given. Pairwise comparisons were performed for good versus poor quality eggs for each time point (0, 14 and 28 dpo), between the time points (0 dpo versus 14 dpo; 0 dpo versus 28 dpo; and 14 dpo versus 28 dpo), as well as across quality and time point groups

Most of transcripts showed dynamic expression pattern across the postovulatory age (Fig. 2). The number of differentially abundant mRNA transcripts within the specific time points and between the two egg quality groups is given in Fig. 3. In total, we found 200 transcripts (< 1%) differentially enriched in pairwise comparisons (Additional file 3). Generally, the differences between egg quality groups (good versus poor quality) in the number of differentially enriched transcripts were not big across the postovulatory period. The dynamics of transcript abundance within the two egg quality groups was similar in the first two weeks of postovulatory age (0 dpo versus 14 dpo), with a significant change in single gene transcript abundance in the poor quality group, and no changes in the good quality group. The significant changes in transcript abundance from 14 to 28 dpo were more pronounced in the poor quality group, whereas the changes from 0 to 28 dpo were more numerous in the good quality group (Fig. 3). However, the differences were higher when cross-comparison between quality groups and between different postovulatory ages was performed.

**Fig. 4:**
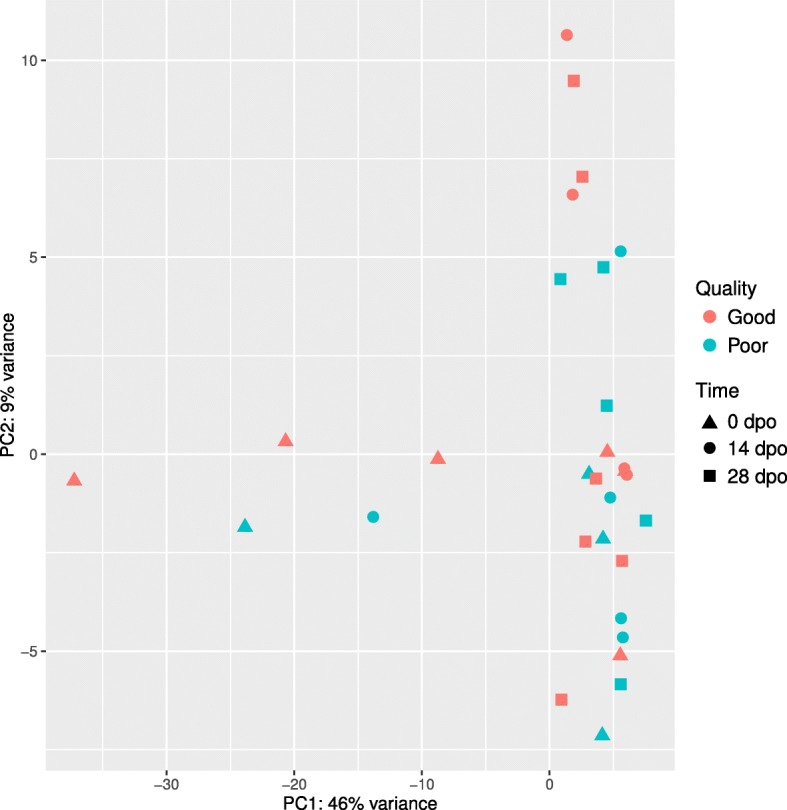
Principal Component Analysis of mRNAs in eggs of Atlantic salmon over the course of postovulatory retention period. Transcripts are projected in the 2-dimensional plane by profile of all transcripts

Transcripts were subjected to k-means clustering, which partitioned them into six clusters, as determined from the within sum squares by the number of clusters plot (Additional file 4). The numbers of transcripts in each cluster were 63, 224, 674, 1485, 3227, and 20,641. Further discriminatory analysis using multiscale bootstrap resampling for each cluster did not entirely reconstruct the original sample groups (Additional file 5). Transcripts abundance for differentially accumulated transcripts, displayed as heat maps in Additional file 6, did not explain the original groupings. Among differentially accumulated transcripts, 158 transcripts had hits for GO term annotation. We found enriched GO terms for some of differentially expressed transcripts (Additional file 7). All samples were examined using principal component analysis (PCA), which showed similar transcript accumulations between good and poor quality eggs (Fig. 4).

### mRNA abundance in egg quality groups in relation to postovulatory age

Majority of differentially accumulated transcripts between the two quality groups (good versus poor) was not informative enough to discriminate the two groups based on transcripts. Most of the differentially accumulated transcripts showed no regularities in expression dynamics between the poor and good egg quality groups throughout the postovulatory retention time (Additional file 3). Those transcripts that consistently showed differential accumulation between the quality groups throughout all the time points were generally low in read counts (Additional file 3). For example, *reticulon-4 receptor-like 2*, *ubiquitin-fold modifier 1*, and *Protein SSUH2 homolog* had consistently higher accumulation in one of the quality groups, but had generally low counts. Transcripts with high read counts, such as *transcription factor IIIB 90 kDa subunit-like*, *signal peptidase complex catalytic subunit SEC11A*, *cathepsin Z-like*, *G0/G1 switch protein 2-like*, *intracellular hyaluronan-binding protein 4*, *pancreatic progenitor cell differentiation and proliferation factor B-like*, *mitogen–activated protein kinase 3-like*, and *acyl-CoA-binding protein*, had inconsistent accumulations between the quality groups and postovulatory ages. Transcripts, such as *NADH dehydrogenase [ubiquinone] 1 alpha subcomplex subunit 8-like*, *WASP homolog-associated protein with actin membranes* and *microtubules-like*, and *phospholipid hydroperoxide glutathione peroxidase mitochondrial* showed higher accumulation in the good quality group at each of the time points. Generally, postovulatory age confounded egg quality in terms of mRNA abundance. Although a number of differentially abundant transcripts could be identified in any given postovulatory time point, both magnitude and relation of transcript levels between the quality groups within a given time point could not be valid when values from different time points were compared. As the outcome, despite a number of candidate transcripts, only one (*hepcidin-1*) was identified as an unbiased indicator of egg quality throughout the postovulatory retention period.

### Testing the potential egg quality markers

**Fig. 5:**
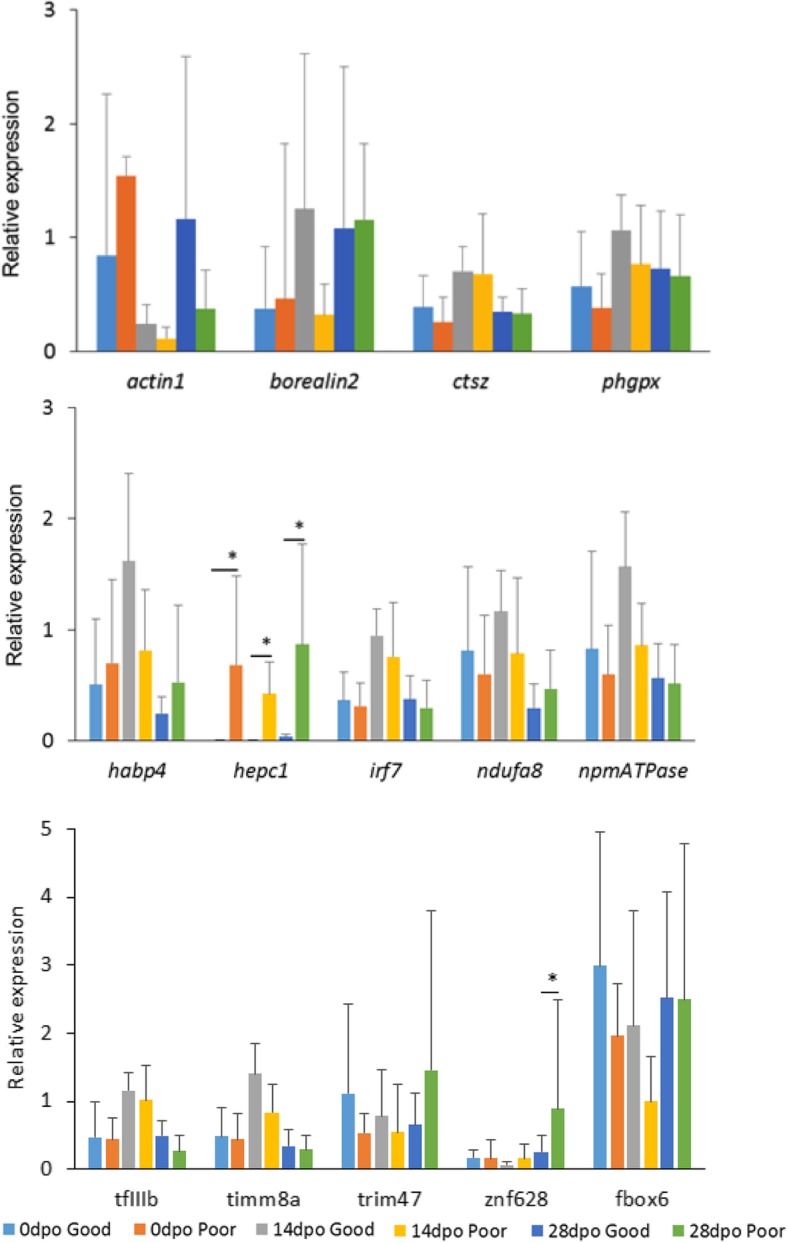
Relative abundance of 14 selected transcripts in Atlantic salmon eggs measured by RT-qPCR method. Expression level (average from n = 6 samples with standard deviation bars) relative to exogenous reference, *luciferase*. * Asterisks mark signficant difference at *p* < 0.05

We performed a RT-qPCR experiment for transcripts of 14 genes, which were selected based on the previous reports in other species, as well as some most promising genes identified in the RNA-seq experiment as described above. Two of them, *hepcidin-1* and *zinc finger protein 628-like*, had significant differences in abundance between the two quality groups. No significant interaction of quality and postovulatory age was found. *Hepcidin-1* showed significantly higher abundance in the poor versus good egg quality group, throughout the all post-ovulatory time points (Fig. 5). In the poor egg quality group, the tested 14 genes showed a consistent decrease in transcript abundance with postovulatory age progression. Whereas, in the good egg quality group, higher transcript abundance was observed at 14dpo compared to 0dpo and 28dpo (Additional file 3).

In another experiment, we further examined the applicability of *hepcidin-1* as a potential egg quality marker using different batches of eggs obtained from other 10 females. Five batches were classified as poor egg quality group and they were characterized by low fertilization (27–61%) and low eyed embryo rates (24–55%), whereas the remaining five batches were considered as good egg quality group, with fertilization and eyed embryos rates at 95–98% and 74–75%, respectively (Additional file 8). We performed RT-qPCR for 8 individual eggs from each batch. A significant difference was found between the two egg quality groups (t-test = − 2.622, *p* = 0.01; Additional file 9).

### Differential abundance of miRNAs

**Fig. 6:**
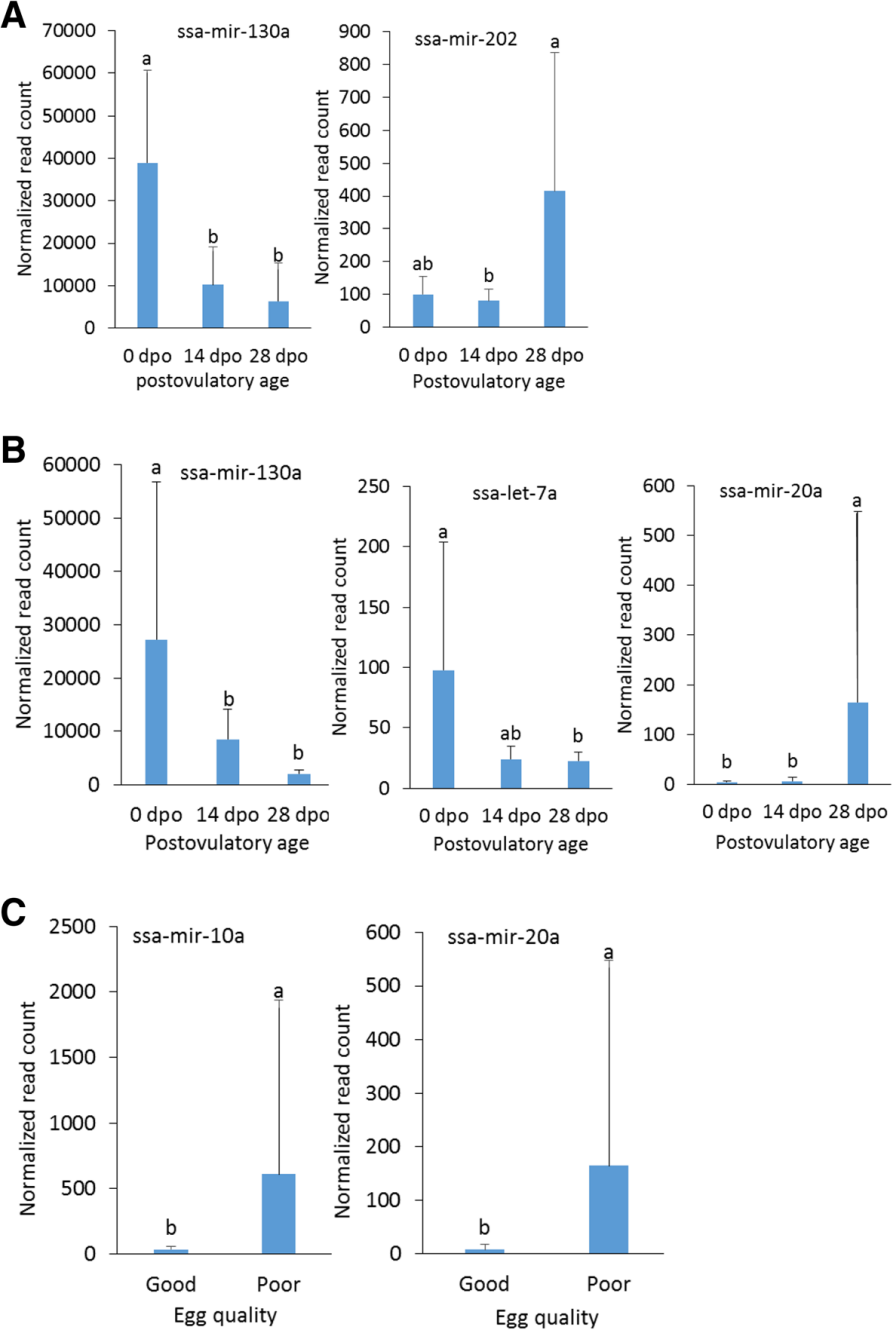
Diffirentially abundant miRNAs in Atlantic salmon eggs throughout the postovulatory retention period. **a**), good egg quality group; **b**), poor egg quality group; **c**), good versus poor quality eggs at 28 days post ovulation (dpo). Normalization is described in Materials and Methods. The error bar is standard deviation. Different letters indicate significant difference at *p* < 0.05

We identified 125 miRNA families in unfertilized egg of Atlantic salmon (Additional file 10). The top most abundant mature miRNAs were ssa-miR-130a, ssa-miR-203a, and ssa-miR-8160. We found five differentially abundant miRNAs (ssa-let-7a, ssa-miR-10a, ssa-miR-20a, ssa-miR-130a, and ssa-miR-202) between the two quality groups, or among the three postovulatory ages within a quality group (Fig. 6). Ssa-miR-130a, which showed differential abundance in relation to the egg quality group, had lower abundance at 28 dpo compared to 0 dpo (FC = − 2.9, FDR = 0.05; FC = − 3.8, FDR < 0.0008, for good and poor egg quality groups, respectively, Additional file 11).

### Maternal miRNA targets

We found 13,365 Atlantic salmon ESTs with 3’UTR sequences. None of the differentially abundant transcripts was found in this set. On average, 67.5 and 72.3 targets were predicted for all identified miRNAs and differentially accumulated miRNAs, respectively.

## Discussion

**Fig. 7:**
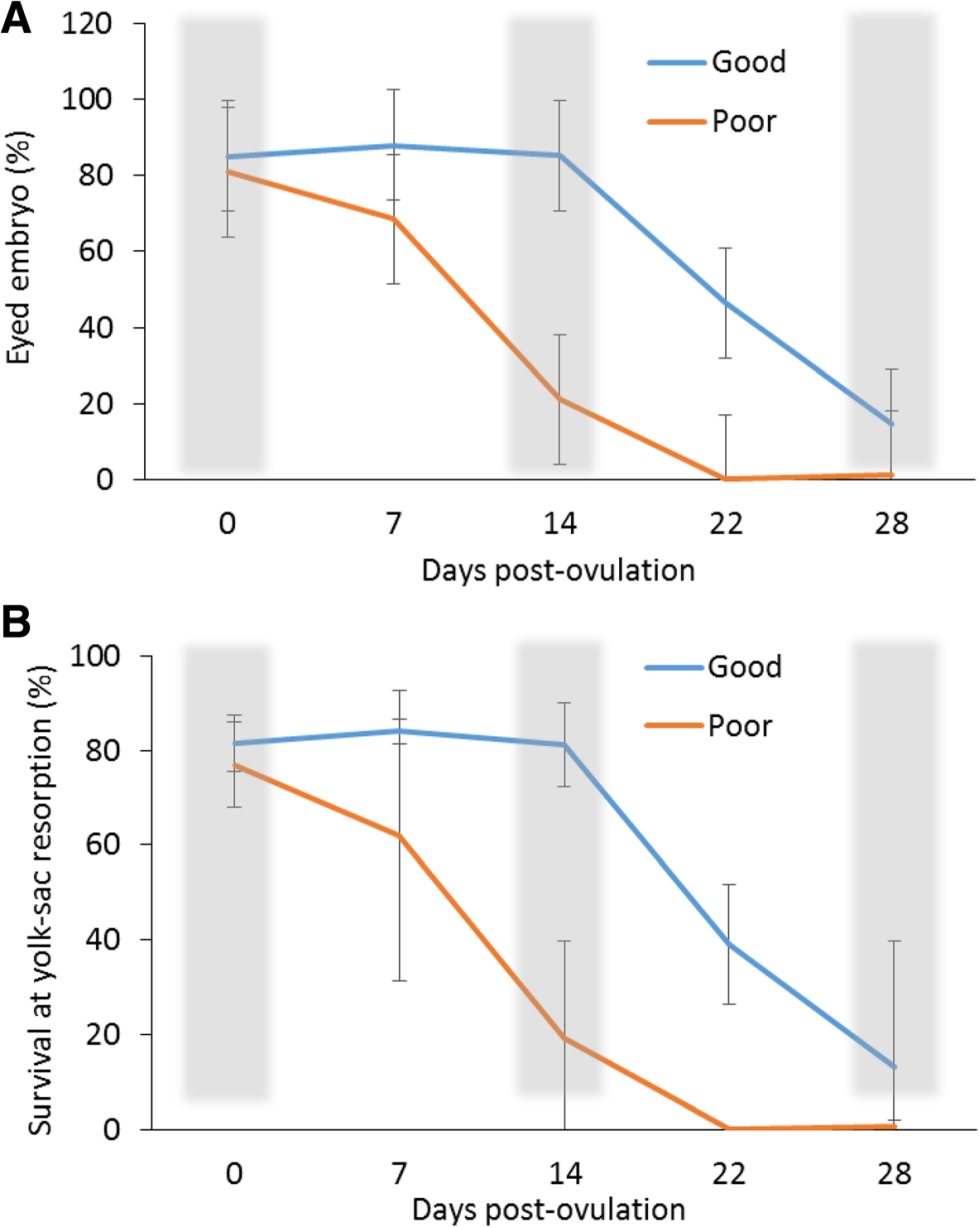
A posteriori developmental performance of Atlantic salmon embryos obtained from good and poor egg quality groups (n = 6 for each). **a**) Eyed embryos; **b**) survival at yolk-sac resorption. RNA-seq data were obtained from three postovulatory ages (shaded). The full experimental set-up is given at Mommens et al. [10]

Postovulatory retention period can be associated with progressing deterioration in egg quality, affecting the developmental competence of a future embryo. In the experiment from which the material was derived, we found that a substantial deterioration in egg quality in Atlantic salmon occurs after 3 weeks of postovulatory retention on average; however, it varies among particular breeders ^8^. The postovulatory loss of developmental competence in time is hypothesized to be caused or at least associated with changes in maternally stocked transcriptome [9, 19]. In this report, we analyzed mRNA and miRNA transcriptomes in batches of eggs during postovulatory retention period, and additionally quantified as of either good or poor quality, based on the results of their developmental potential, reported previously ^8^. The dynamics of decrease in transcript abundance, observed in the good egg quality group between the start (0 dpo) and the end (28 dpo) of the postovulatory retention period (Fig. 3), was not associated with the deterioration in developmental competence, which started from 14 dpo onwards (Fig. 7); in this period (14 dpo to 28 dpo), deterioration in transcript abundance was incidental, if any (Fig. 3). Moreover, no distinctive transcripts accumulation pattern was found between the good versus poor quality egg groups by clustering and principal component analyses. Clusters with mixed samples from the two quality groups (Fig. 4, Additional file 5) reflect the advancement in postovulatory processes rather than the deterioration in egg quality itself. High individual variation could contribute for lack of the original grouping in cluster analysis due to small sample size. Transcriptome of poor quality eggs had different dynamics in time than in the good quality group.

Some mRNAs found in our dataset have been associated with egg quality in different fish species. They include *insulin-like growth factor 1*, *insulin-like growth factor 2*, *insulin-like growth factor receptor*, *tubulin*, *apolipoprotein C1*, *cyclin B*, *keratin*, *amidohydrolase 1*, *prohibitin 2*, *prostaglandin synthase 2*, *pyruvate carboxylase*, and *60S ribosomal protein L24* identified in rainbow trout [9]; *cytochrome p450*, *Exportin-1-like, Major histocompatibility complex class ii antigen alpha chain*, *programmed cell death 1 ligand*, and *ring finger protein 213* reported in Atlantic halibut [20]; and *3-hydroxyacyl-CoA dehydratase 1*, *importin subunit alpha*, *aromatic-L-amino-acid-decarboxylase*, *discoidin, CUB and LCCL domain containing 1*, *glutathione peroxidase*, and *heat shock protein 70* found in Atlantic cod [21]. However, none of these transcripts was differentially accumulated between the good and poor quality eggs in the current study. We also tested some of these gene transcripts using the RT-qPCR method, but we did not find *cathespsin Z* [9] and *interferon regulatory factor 7* [20] as potential markers of egg quality in Atlantic salmon (Fig. 5). These dissimilarities can come from species-specific difference in egg transcriptome accumulation. Alternatively, subtle differences in cohort of transcripts could determine egg quality rather than a higher accumulation or in single gene transcripts [12]. Thus, further comparative studies are needed to identify conserved patterns in different teleosts.

Maternally stocked transcripts have different levels of abundance during the oocyte maturation, activation, and maternal-zygote transition [16]. In *Xenopus laevis*, postovulatory aging of oocytes resulted in mRNA deadenylation [22]. In general, transcript abundance in the present study has decreased towards the end of postovulatory retention period, likely because of autonomous degradation processes. As this postovulatory aging-related degradation shows no strong effect on egg quality, its occurrence could be related to clearance of oocyte maturation transcripts, redundant for further developmental competence, rather than to temporarily essential transcriptome.

The role of miRNA in postovulatory aging is unknown. We found diverse types of miRNAs in Atlantic salmon oocytes, in agreement to previous study in rainbow trout [23]. Recent study on rainbow trout oocytes with three postovulatory ages (1, 7, and 14 dpo) and two quality groups (“bad” and “good”) has shown a significant decrease in six miRNAs at 14 dpo compared to 1 and 7 dpo [18]. Mature oocytes are translationally quiescent [16]. The major protein component of miRNA-induced silencing complex (miRISC), AGO PIWI domain, is present in a limited amount in zebrafish oocytes [24]; however, to our knowledge, no study has shown any post-transcriptional regulation in metaphase II-arrested oocytes. It has been reported that miRNAs lack regulatory modulation of their target mRNAs in mouse oocytes [25]. Although maternally provided miRNAs have regulatory role during embryogenesis; the relevance of the differential accumulation of miRNAs in mature oocytes is unclear.

The increase in transcript levels in later postovulatory age, observed in both mRNA and miRNA datasets, needs further investigation. The current knowledge is that after intensive transcriptional activity during the growth stages, mature oocytes, arrested in the metaphase of the second meiotic division, are transcriptionally silent [16]. However, post-transcriptional processing can occur, as demonstrated in *Xenopus* [26]. It is unknown whether it occurs to native primary transcripts in fish oocytes. Alternatively, the increase in levels of some transcripts could be related to inherent heterogeneity of eggs. Each egg represents a unique entity, and samples of eggs from the same female are not identical in transcriptome composition. In addition, Atlantic salmon ancestors in vertebrate lineage had undergone several rounds of the whole genome duplication, resulting in multiple ohnologs and possible transcript isoforms [27]. It creates ambiguity in assigning reads to their transcript of origin and obtaining precise counts and can potentially bias the proportions between the observed transcript levels.

Although a number of mRNAs showed differential abundance between time points and/or egg quality groups (200 transcripts), their application as egg quality markers would be difficult. The majority of differentially abundant transcripts was detectable in both egg quality groups, in levels varying highly in relation to postovulatory age. Therefore, establishing a threshold expression level for good or poor eggs would be affected by the postovulatory age of a given egg batch. *Hepcidin 1* was the single exception, as it accumulated in poor quality eggs but not in good quality eggs, regardless of postovulatory age (Fig. 5 and Additional file 9A). Hepcidin is known as an antimicrobial peptide in different species, including Atlantic salmon [28]. Different types of hepcidin-like peptides have been characterized in Atlantic salmon development; they showed tissue-specific expression pattern and increased expression during bacterial infection [29]. Apart from its role in pathogen defense, Hepcidin has been suggested to have a role in iron homeostasis in sea bass (*Dicentrarchus labrax*) [30]. Thus, the higher abundance of *hepcidin* in the poor quality eggs, observed in this study, could be associated with the stress conditions. With further test on eggs from other 10 females, we demonstrated that *hepcidin-1* has a potential as egg quality marker. Simpler markers, such as ovarian fluid pH, morphological features of an egg and blastomere morphology [11, 31, 32] have some degree of applicability. However, in a practical sense, both morphological and genetic-based methods have limitations as predictors of oocyte developmental competence.

## Conclusions

Analysis of mRNA and miRNA transcriptomes from Atlantic salmon oocytes during postovulatory retention period of 4 weeks revealed that (1) dynamics of transcriptome during postovulatory retention in good egg quality group differs from poor egg quality group; (2) there is no apparent relation of transcriptome repertoire and egg quality mostly because of confounding effect of postovulatory age; (3) *hepcidin-1* can be a potential egg quality marker for Atlantic salmon.

## Methods

### Animals and gamete collection

All experimental procedures and animal handling were according to the guidelines of Norwegian regulation for laboratory animal experimentation (The Norwegian Animal Protection Act, No. 73 of 20 December 1974, Section 20–22, amended 19 June 2009).

The details about the animals and sampling are given in Mommens et al. [10]. Briefly, fish originated from and were handled at Aqua Gen AS facility (Trondheim, Norway). Eggs were collected by hand stripping from the same females at three time points of egg retention period. A portion of 500–600 eggs was stripped from every female every second week starting from the onset of ovulation (day 0 post-ovulation, 0 dpo), 14 dpo, and 28 dpo. The reason of selection of these time-points was that oocyte retention in body cavity of salmonid fishes lasts for approximately a month [10], therefore the selected time-points secured the early, medium, and advanced stages of oocyte retention, respectively. A part of each portion of eggs was snap frozen in liquid nitrogen, and the remaining eggs were fertilized with a cryopreserved sperm from the same single male [10]. Based on fertilization rates, hatching success, and larval development performances [10], batches of eggs from 12 out of 20 females were selected for the analyses. They were categorized into two groups (*n* = 6 each) representing good versus poor quality eggs (Fig. 7). Altogether, 36 egg batches (12 females × 3 time points) were selected for sequencing.

### RNA extraction and sequencing

We performed all RNA extraction, library preparation and sequencing at the Nord University, Bodø, Norway. Total RNA was extracted from 20 eggs from each egg batch using Trizol reagent (Invitrogen) and RNeasy purification kit (QIAGEN). The quality of RNA was checked using bioanalyzer (Agilent Technologies, Waldbronn, Germany) and RNA with RIN > 7.5 were used for sequencing library preparation. For mRNA-seq, ribosomal RNAs were depleted using RiboMinus Eukaryote Kit v2 (Life Technologies). Sequencing libraries were prepared using SOLiD Whole Transcriptome Analysis Kit (Life Technologies) according to the manufacturer protocol. Sequencing was performed with sequence length of 75 nucleotides (nts) on six lanes of a slide. For small RNAs, libraries were prepared using total RNA following SOLiD Whole Trascriptome Analysis Kit small RNA protocol, and sequenced with read length of 35 nts on three lanes of a slide. Sequencing was performed on 5500xl SOLiD sequencer (Life Technologies, Tokyo, Japan).

### Data analysis

Raw reads were processed using cutadapt v1.4.1 [33] to remove adapters and low quality reads. These sequences were aligned against External RNA Controls Consortium (ERCC Spike-In Control Mixes, Ambion) using bowtie2 v2.2.3 [34] for library prep quality control. After quality control, cleaned RNA-seq data were aligned using TopHat2 v2.0.11 [35] using Atlantic salmon genome and its reference gene annotation GTF [36]. Reads mapped to each transcript were counted using featureCounts v1.4.6. [37]. Gene ontology (GO) terms were assigned by blasting the annotated transcripts to GO database [38]. Enrichment analysis was performed using topGO an R package [39] using differential accumulated transcripts GO terms and all annotated GO terms as a background. Fisher exact test was used, and *p*-value < 0.01 was considered as a significance threshold.

Small RNA analysis was performed after trimming adapter sequences using cutadapt [33]. miRNAs were annotated using miRDeep2 [40] using Atlantic salmon miRNAs from miRBase21 [41].

### Differential abundance analysis

Differential abundance analysis of mRNA transcripts was performed using EdgeR with upper quintile normalization [42]. Explanatory analysis of the mRNA-seq data identified one sample (poor quality group at 0 dpo) contributing 5% of the total biological coefficient of variation observed in the whole dataset. In addition, this sample had less transcriptome diversity, as well as fewer types of transcripts with high number of reads compared to other samples in the group. The discreteness of these counts interfered with some of the statistical approximations that were used in EdgeR. Therefore, we removed this sample from differential accumulation analysis.

Differential abundance of miRNA transcripts was assessed using EdgeR. To separate noise from signal in low-count reads, we included putative miRNA into analysis when it had representation in minimum four samples per quality/age group. We used general linear model with time and quality as main factors and their interaction. Statistical comparisons were performed between “good” and “poor” egg quality groups, among sampling time points, and their combination. Benjamini and Hochberg’s procedure was used for multiple testing correction to obtain adjusted false discovery rate (FDR). FDR < 0.05 was considered as significant.

### Cluster analysis

Differentially abundant mRNA transcripts were partitioned according to their abundance pattern by the k-means clustering method in the R statistical package (version 3.1.1). The number of clusters was determined by the plot of the within sum of squares by number of clusters. To support the clustering with statistics, sample clustering was done using pvclust in the R statistical package, which assesses the uncertainty in hierarchical cluster analysis by assigning *p*-values through multiscale bootstrap resampling. We used Ward D cluster method, average distance, and 10,000 bootstraps.

### miRNA target prediction

We downloaded the Atlantic salmon Expressed Sequence Tags (ESTs) from National Center for Biotechnology Information (NCBI) and extracted the 3′ UTR sequences. miRNA target prediction was performed using Miranda [43] with strict seed match, free energy < − 20 kcal/mol, and the pairing score > 150.

### Real-time quantitative reverse transcription PCR (RT-qPCR)

Primers were designed for selected transcripts from our RNA-seq, as well as gene transcripts that have been associated with egg quality in previous studies [12] (Additional file 12). Complementary DNA (cDNA) was synthesized from 250 ng of total RNA using QuantiTect Reverse Transcription Kit (Qiagen). For each sample, 10 pg luciferase RNA was added during the reverse transcription. The cDNA was diluted 60 times and 4 μl volume was used as a template for real time PCR. The expression levels of transcripts were quantified using StepOnePlus PCR System (Applied Biosystems, Singapore). The PCR reactions were set up in transparent 96-well plates with 10 μl reaction volume. The thermal cycling conditions included 40 cycles of 95 for 3 s, 60 for 30 s, and 72 for 15 s. Relative expression was calculated by dividing the relative expression of a gene of interest with that of the relative expression of exogenous reference gene (luciferase). The effects of egg quality (good versus poor) and postovulatory age (0 dpo, 14 dpo, and 28 dpo) were estimated using ANOVA. All statistical analyses were performed in R [44]. *P*-value < 0.05 was considered as significant.

## Additional files

Additional file 1: Overview of raw, cleaned, and annotated reads obtained from mRNA-seq of unfertilized eggs of Atlantic salmon. (DOCX 97 kb)

Additional file 2: Blast results of transcripts from Atlantic salmon eggs. Transcript length, e-value, Refseq ID, GO-terms ID and GO-terms are given. The GO-term categories are cellular components (C), molecular function (F), and biological process (P). (XLS 19155 kb)

Additional file 3: Differentially accumulated transcripts from Atlantic salmon eggs and their annotation. (XLS 119 kb)

Additional file 4: Plot of the optimum number of clusters for k-means clustering of all differentially accumulated transcripts determined by within groups sum of squares by number of clusters. The plot determines six clusters (red arrow) as the best solution to partition the differentially accumulated transcripts. (PDF 16 kb)

Additional file 5: Cluster dendrogram for differentially abundant mRNAs in Atlantic salmon eggs from good versus poor egg quality groups, over the course of postovulatory retention period. The uncertainty in hierarchical cluster analysis was performed by pvclust using average distance method in R statistical package. *P*-values of clusters are assigned for each branch. The red is approximately unbiased (AU), the green is bootstrap probability (BP); 10,000 bootstrap iterations were used. (PDF 116 kb)

Additional file 6: Heatmaps of differentially accumulated mRNAs in eggs of Atlantic salmon over the course of postovulatory retention period. Each row represents a transcript; dark red color represents high accumulation and yellow color represents low accumulation. (PDF 58 kb)

Additional file 7: GO term enrichment. The number of total and differentially accumulated transcripts is shown for enriched GO terms. (XLSX 17 kb)

Additional file 8: RNA concentration and quality scores for sequencing library preparation. RNA quality was measured by bioanalyzer. RNA was extracted using Trizol and RNeasy kit. N/A = not available. (DOCX 22 kb)

Additional file 9: Relative abundance of *hepcidin-1* in Atlantic salmon egg grouped as poor and good quality (*n* = 8) as measured by RT-qPCR method. (DOCX 109 kb)

Additional file 10: miRNA names, sequences and their read counts. (DOCX 72 kb)

Additional file 11: miRNA sequence, count, and stastistic in Atlantic salmon egg from good versus poor egg quality groups (good and poor), over the course of postovulatory retention period (0, 14, 28 day post-ovulation). Each tab contain the statistical results obtained from EdgeR for each comparison. (XLS 489 kb)

Additional file 12: Primers used for RT-qPCR. Gene name and symbol with forward and reverse primer sequences (5′ -3′) are given. (DOCX 19 kb)

## Abbreviations

3′ UTR: 3′ untranslated region
dpo: days post-ovulation
EST: Expressed sequence tag
FC: Fold-change
FDR: False discovery rate
GO: Gene ontology
miRNA: micro RNA
mRNA: messenger RNA
nt: nucleotide
PCA: Principal component analysis
RPKM: Reads per kilobase million
RT-qPCR: Real-time quantitative reverse transcription PCR
