# Supplementary material for: Postovulatory maternal transcriptome in Atlantic salmon and its relation to developmental potential of embryos
Source: BMC Genomics. 2019 Apr 24;20:315. doi: 10.1186/s12864-019-5667-4 (PMC6480738; doi:10.1186/s12864-019-5667-4)
Supplement: Supplementary file 1 — Overview of raw, cleaned, and annotated reads obtained from mRNA-seq of unfertilized eggs of Atlantic salmon. (DOCX 97 kb) [file 12864_2019_5667_MOESM1_ESM.docx]

Additional file 1. Overview of raw, cleaned, and annotated reads obtained from mRNA-seq of unfertilized eggs of Atlantic salmon.

| Postovulatory age | Quality group | Raw reads | Cleaned reads | Annotated reads |
| --- | --- | --- | --- | --- |
| 0 dpo | Good | 1 653 168 | 1 507 477 | 486 552 |
|  |  | 1 395 473 | 1 106 800 | 343 010 |
|  |  | 26 653 870 | 21 030 457 | 6 588 420 |
|  |  | 22 155 057 | 18 142 281 | 5 500 125 |
|  |  | 21 364 440 | 17 190 788 | 5 272 943 |
|  |  | 23 768 516 | 18 065 409 | 5 064 423 |
|  | Poor | 15 833 832 | 12 382 927 | 2 790 153 |
|  |  | 24 426 072 | 18 050 908 | 3 165 031 |
|  |  | 23 305 113 | 19 374 701 | 6 513 665 |
|  |  | 16 635 379 | 12 202 434 | 2 869 913 |
|  |  | 48 425 412 | 41 446 193 | 12 580 652 |
| 14 dpo | Good | 31 577 994 | 27 695 201 | 7 435 651 |
|  |  | 20 329 334 | 16 959 225 | 4 347 399 |
|  |  | 23 109 899 | 19 444 733 | 5 082 736 |
|  |  | 30 254 615 | 25 865 398 | 6 533 422 |
|  | Poor | 18 257 214 | 14 549 312 | 4 121 737 |
|  |  | 14 952 345 | 12 833 238 | 3 249 432 |
|  |  | 18 822 511 | 15 137 979 | 3 257 363 |
|  |  | 29 621 822 | 26 039 026 | 7 181 983 |
|  |  | 29 773 090 | 25 356 883 | 7 227 588 |
| 28 dpo | Good | 22 720 660 | 17 860 307 | 4 072 301 |
|  |  | 29 132 168 | 25 800 061 | 7 795 194 |
|  |  | 15 554 345 | 12 125 239 | 3 190 871 |
|  |  | 16 251 597 | 12 215 722 | 3 237 457 |
|  |  | 20 764 821 | 18 227 016 | 4 694 471 |
|  |  | 15 012 062 | 12 607 540 | 3 617 456 |
|  | Poor | 16 046 416 | 12 663 214 | 3 485 515 |
|  |  | 12 179 813 | 10 416 836 | 2 476 212 |
|  |  | 19 626 854 | 16 718 209 | 4 350 850 |
|  |  | 11 786 379 | 9 649 780 | 2 619 308 |
|  |  | 18 886 697 | 16 716 978 | 5 341 278 |
| Total reads | | 640 276 968 | 529 382 272 | 144 493 111 |
