# Supplementary figures and images for: Postovulatory maternal transcriptome in Atlantic salmon and its relation to developmental potential of embryos
Source: BMC Genomics. 2019 Apr 24;20:315. doi: 10.1186/s12864-019-5667-4 (PMC6480738; doi:10.1186/s12864-019-5667-4)

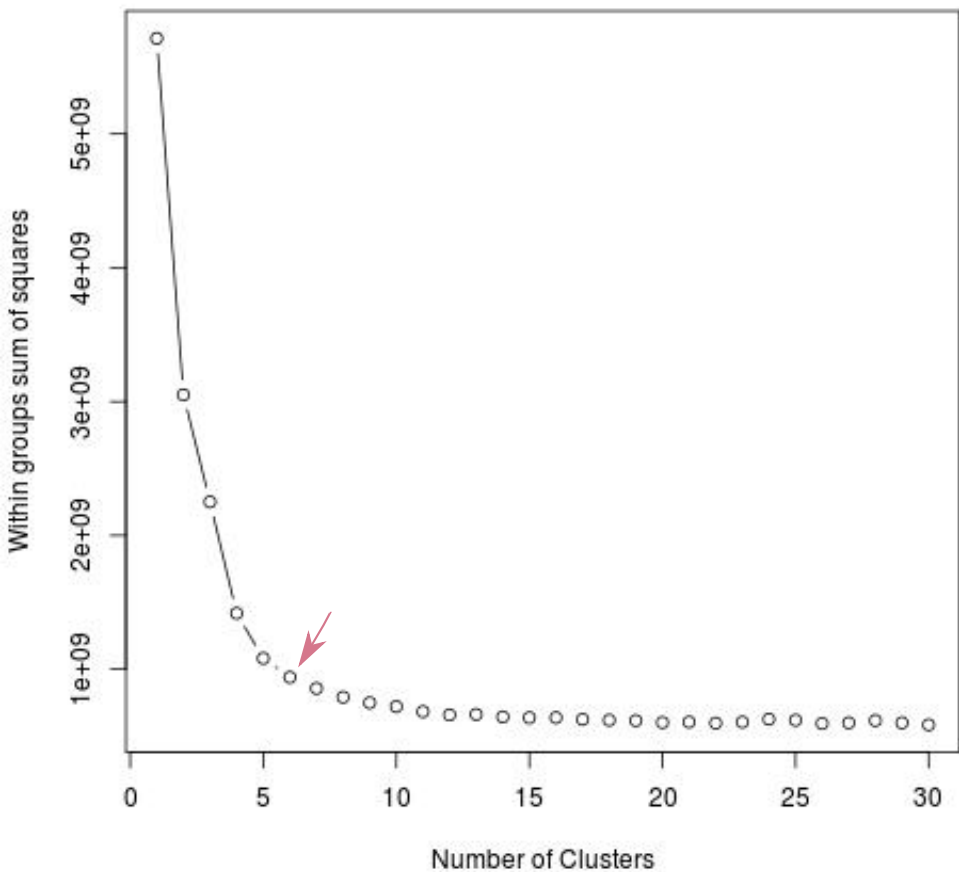

Supplement: Supplementary file 4 — Plot of the optimum number of clusters for k-means clustering of all differentially accumulated transcripts determined by within groups sum of squares by number of clusters. The plot determines six clusters (red arrow) as the best solution to partition the differentially accumulated transcripts. (PDF 16 kb) [file 12864_2019_5667_MOESM4_ESM.pdf]

# Cluster 1

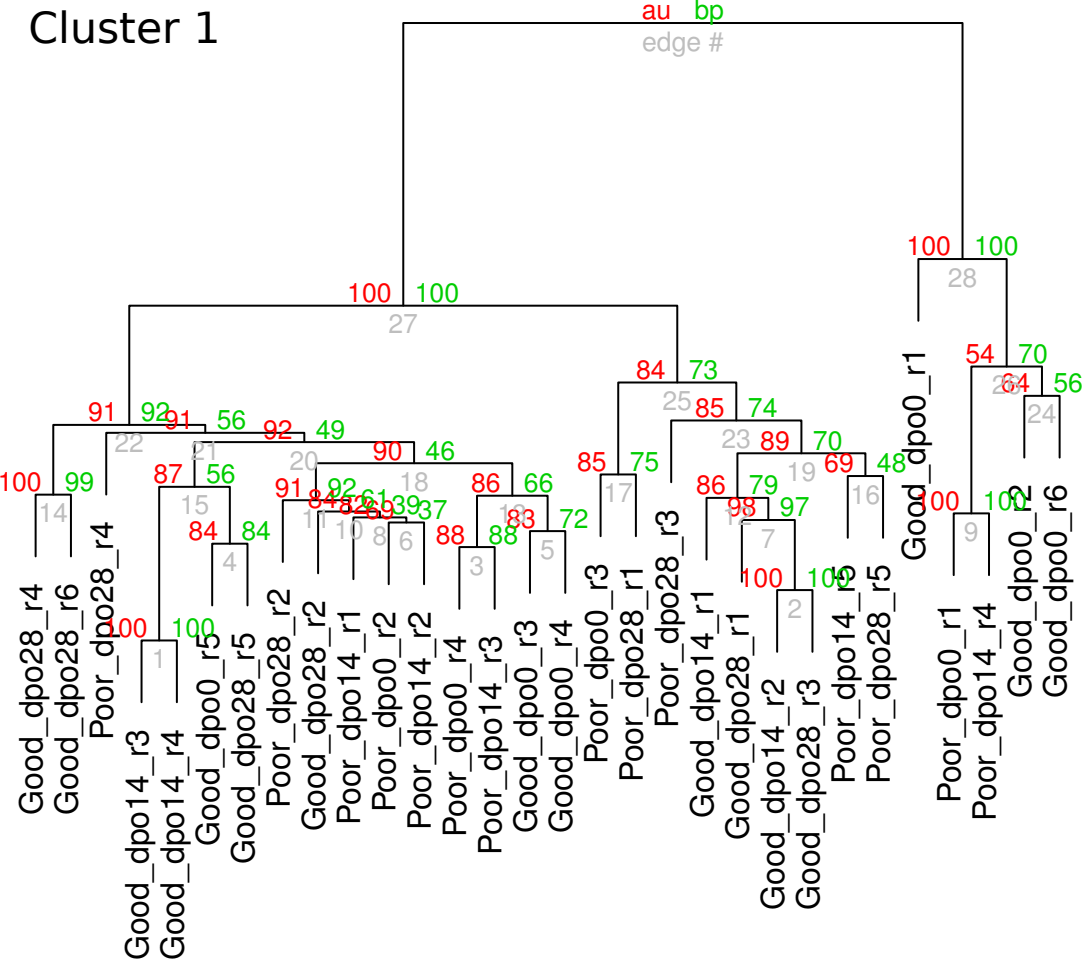

au bp

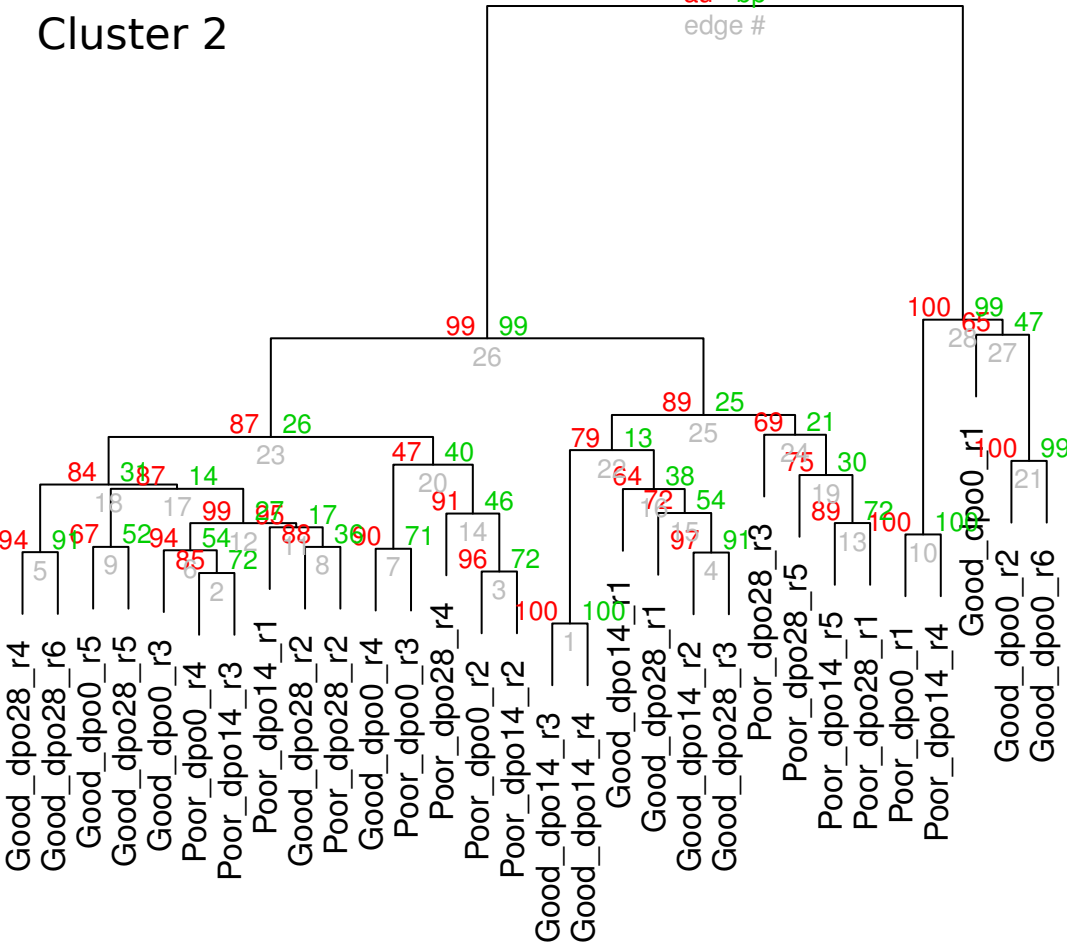

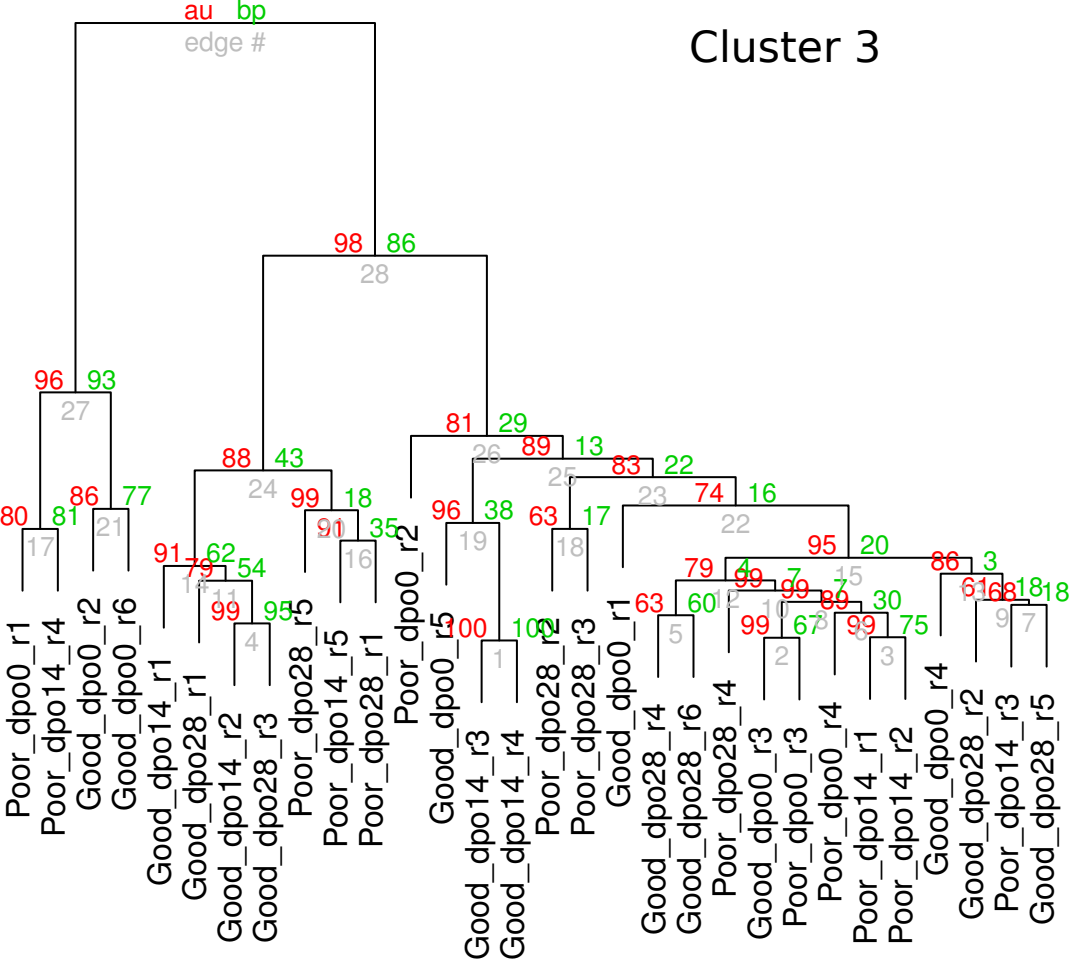

## Cluster 4

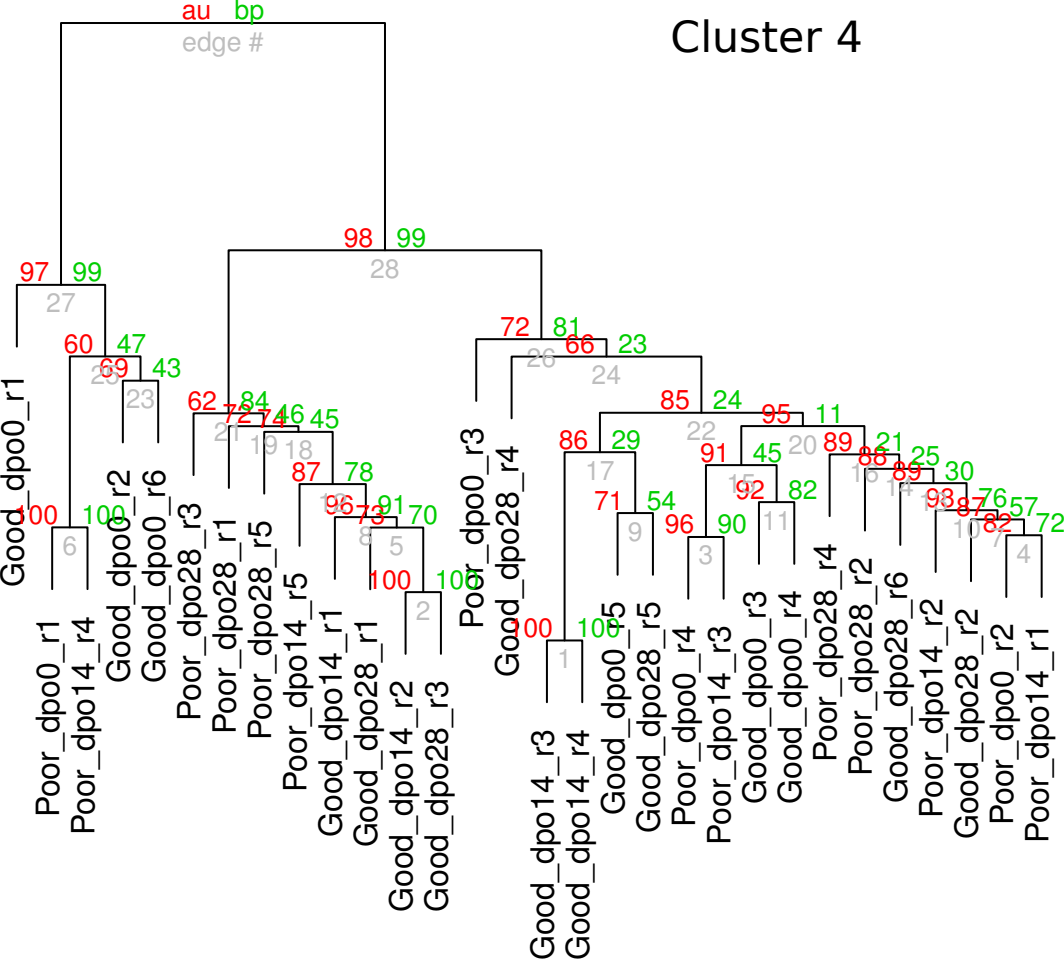

# Cluster 5

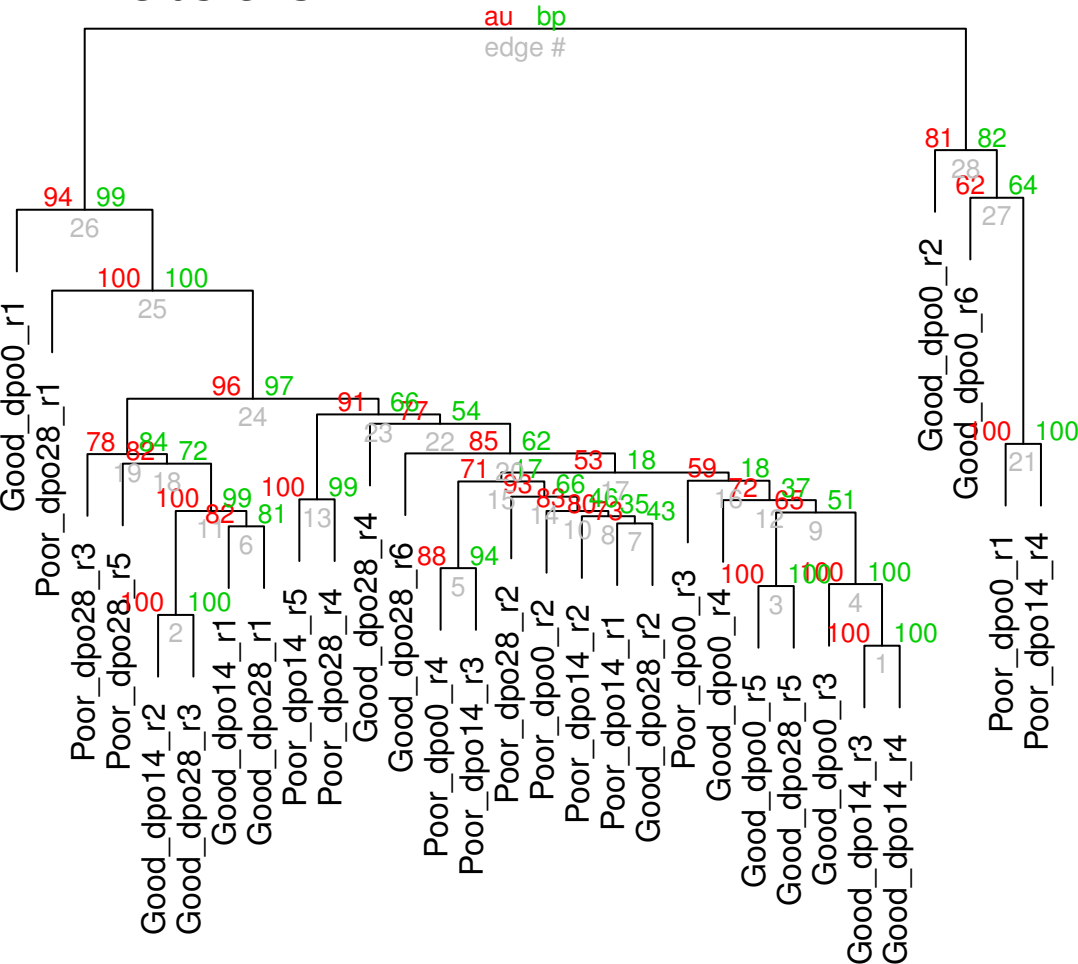

# Cluster 6

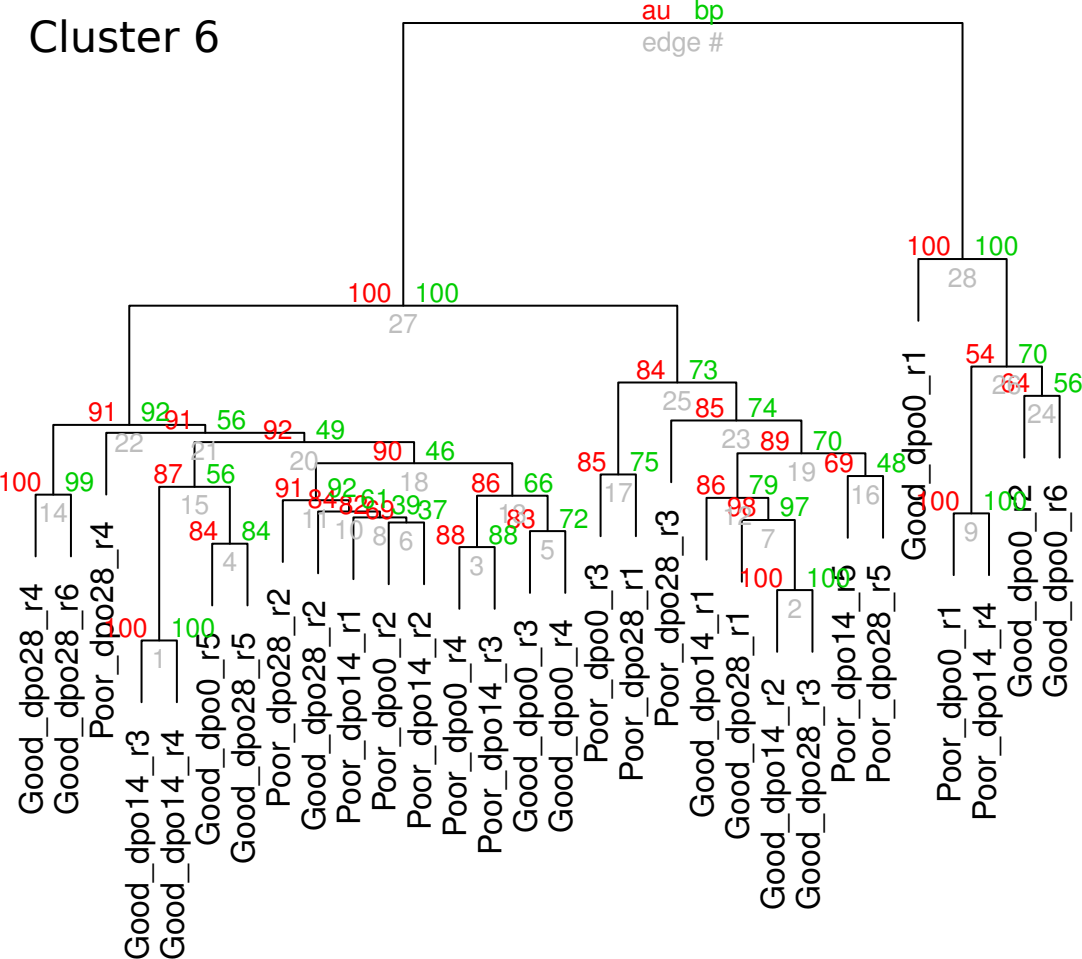

Supplement: Supplementary file 5 — Cluster dendrogram for differentially abundant mRNAs in Atlantic salmon eggs from good versus poor egg quality groups, over the course of postovulatory retention period. The uncertainty in hierarchical cluster analysis was performed by pvclust using average distance method in R statistical package. P-values of clusters are assigned for each branch. The red is approximately unbiased (AU), the green is bootstrap probability (BP); 10,000 bootstrap iterations were used. (PDF 116 kb) [file 12864_2019_5667_MOESM5_ESM.pdf]

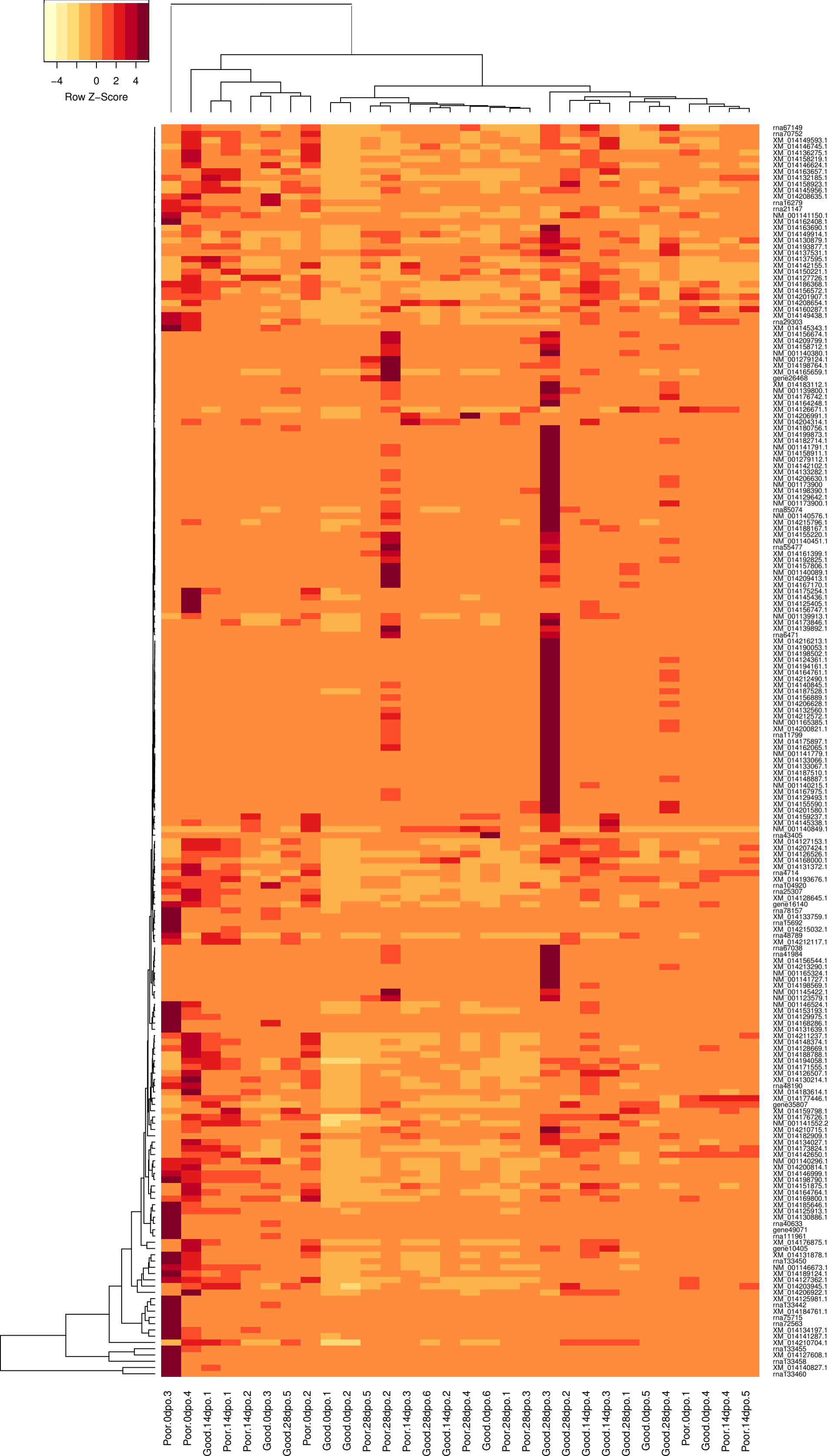

Supplement: Supplementary file 6 — Heatmaps of differentially accumulated mRNAs in eggs of Atlantic salmon over the course of postovulatory retention period. Each row represents a transcript; dark red color represents high accumulation and yellow color represents low accumulation. (PDF 58 kb) [file 12864_2019_5667_MOESM6_ESM.pdf]
