# Supplementary material for: Postovulatory maternal transcriptome in Atlantic salmon and its relation to developmental potential of embryos
Source: BMC Genomics. 2019 Apr 24;20:315. doi: 10.1186/s12864-019-5667-4 (PMC6480738; doi:10.1186/s12864-019-5667-4)
Supplement: Supplementary file 8 — RNA concentration and quality scores for sequencing library preparation. RNA quality was measured by bioanalyzer. RNA was extracted using Trizol and RNeasy kit. N/A = not available. (DOCX 22 kb) [file 12864_2019_5667_MOESM8_ESM.docx]

| Additional file 8. RNA quality as measured by bioanlyzer. Rna extracted using trizol and RNeasy kit | | | | | | |  |
| --- | --- | --- | --- | --- | --- | --- | --- |
| Postovulatory-days | Female ID | Sample ID | Quality group | Trizol [ng/ul] | RIN | RNeasy kit (ng/ul) | RIN |
| 0 | 427 | 2 | good | 75 | 9 | 900 | 10 |
| 0 | 432 | 7 | good | 130 | 9 |  |  |
| 0 | 437 | 12 | good | 57 | 9,5 | 143 | 9,9 |
| 0 | 438 | 13 | good | 50 | N/A | 120 | 10 |
| 0 | 445 | 19 | good | 36 | 9,8 | 449 | 10 |
| 0 | 446 | 20 | good | 68 | N/A | 1536 | 10 |
| 0 | 429 | 4 | low | 81 | 8,5 | 595 | 9,8 |
| 0 | 431 | 6 | low | 97 | 9,2 | 212 | 10 |
| 0 | 434 | 9 | low | 52 | N/A | 102 | 7,6 |
| 0 | 440 | 15 | low | 69 | 8,9 | 542 | 10 |
| 0 | 447 | 23 | low | 53 | 9,1 | 1200 | 10 |
| 0 | 448 | 30 | low | 78 | 9,5 | 159 | 10 |
| 14 | 427 | 42 | good | 67 | N/A | 157 | 9,7 |
| 14 | 432 | 47 | good | 67 | 9,5 | 244 | 10 |
| 14 | 437 | 52 | good | 52 | N/A | 322 | n/a |
| 14 | 438 | 53 | good | 35 | N/A | 571 | 10 |
| 14 | 445 | 59 | good | 58 | N/A | 1238 | 10 |
| 14 | 446 | 60 | good | 72 | 9,3 |  |  |
| 14 | 429 | 44 | low | 42 | N/A | 182 | 9,7 |
| 14 | 431 | 46 | low | 61 | N/A | 716 | 10 |
| 14 | 434 | 49 | low | 27 | N/A | 367 | 9,2 |
| 14 | 440 | 55 | low |  |  | 62 | n/a |
| 14 | 447 | 43 | low | 38 | N/A | 341 | 9,9 |
| 14 | 448 | 50 | low | 68 | N/A | 180 | n/a |
| 28 | 427 | 82 | good | 53 | N/A | 212 | 10 |
| 28 | 432 | 87 | good | 62 | 9,4 | 311 | 9,8 |
| 28 | 437 | 92 | good | 51 | 10 | 317 | 9,6 |
| 28 | 438 | 93 | good | 50 | N/A | 1026 | 9,8 |
| 28 | 445 | 99 | good | 53 | N/A | 879 | 9,7 |
| 28 | 446 | 100 | good | 62 | 9,8 | 203 | 9,9 |
| 28 | 429 | 84 | low | 68 | 7,8 | 358 | 10 |
| 28 | 431 | 85 | low | 55 | 9,6 | 375 | 9,5 |
| 28 | 434 | 89 | low | 69 | 7,9 | 171 | 9,9 |
| 28 | 440 | 95 | low | 64 | 9,1 | 142 | 9 |
| 28 | 447 | 83 | low | 67 | 10 | 75 | 10 |
| 28 | 448 | 90 | low | 28 | N/A | 767 | 9,9 |
