# Supplementary material for: Postovulatory maternal transcriptome in Atlantic salmon and its relation to developmental potential of embryos
Source: BMC Genomics. 2019 Apr 24;20:315. doi: 10.1186/s12864-019-5667-4 (PMC6480738; doi:10.1186/s12864-019-5667-4)
Supplement: Supplementary file 9 — Relative abundance of hepcidin-1 in Atlantic salmon egg grouped as poor and good quality (n = 8) as measured by RT-qPCR method. (DOCX 109 kb) [file 12864_2019_5667_MOESM9_ESM.docx]

**Additional file 9**

Mean relative expression of *hepcidin-1* as determined by qPCR from salmon egg grouped as poor and good quality (see below). Error bar indicate standard deviation and *indicates p-value < 0.01.

A.


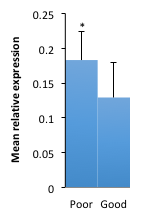


B.

qPCR experiment was performed using 10 individual females. Their and 15 other females performance data is depicted in the Table below. From each female 3x200 eggs were fertilized with sperm from one male. Fertilization percentage analysis was performed at 24h after fertilization (120 degree-day). Fertilization % is based on 20 eggs from each triplicate. Eggs were shocked at 319 d° and white eggs removed. Eyed % was estimated at 354 d° on the remaining eggs after shocking. **Poor egg quality group were characterized by low fertilization (27 – 61 %) and low eyed embryo rates (24 – 55 %), whereas good egg quality group had fertilization and eyed embryos rates at 95 – 98 % and 74 – 75 %, respectively.**

| **Female ID** | **Fertilization % at 120** d° | **STD** | **Eyed %** | **STD** | **selected** |
| --- | --- | --- | --- | --- | --- |
| 126 | 78 | 4 | 66 | 1 |  |
| 127 | 93 | 3 | 72 | 1 |  |
| 128 | 72 | 8 | 60 | 2 |  |
| 129 | 82 | 3 | 67 | 3 |  |
| 130 | 61 | 6 | 55 | 5 | Poor |
| 131 | 88 | 3 | 63 | 1 |  |
| 132 | 90 | 1 | 53 | 4 |  |
| 133 | 91 | 3 | 72 | 6 |  |
| 134 | 60 | 7 | 39 | 9 | Poor |
| 135 | 98 | 3 | 75 | 1 | Good |
| 136 | 68 | 4 | 65 | 4 |  |
| 137 | 80 | 7 | 61 | 5 |  |
| 138 | 80 | 5 | 58 | 3 |  |
| 139 | 27 | 0 | 24 | 4 | Poor |
| 140 | 97 | 6 | 77 | 1 | Good |
| 141 | 92 | 4 | 73 | 4 |  |
| 142 | 77 | 7 | 70 | 3 |  |
| 143 | 95 | 5 | 75 | 1 | Good |
| 144 | 56 | 5 | 47 | 5 | Poor |
| 145 | 90 | 7 | 69 | 1 |  |
| 146 | 95 | 5 | 74 | 1 | Good |
| 147 | 73 | 0 | 60 | 4 |  |
| 148 | 95 | 0 | 74 | 2 | Good |
| 149 | 80 | 0 | 65 | 2 |  |
| 150 | 53 | 4 | 38 | 4 | Poor |
