# Supplementary material for: Postovulatory maternal transcriptome in Atlantic salmon and its relation to developmental potential of embryos
Source: BMC Genomics. 2019 Apr 24;20:315. doi: 10.1186/s12864-019-5667-4 (PMC6480738; doi:10.1186/s12864-019-5667-4)
Supplement: Supplementary file 10 — miRNA names, sequences and their read counts. (DOCX 72 kb) [file 12864_2019_5667_MOESM10_ESM.docx]

| Additional file 10. miRNA names, sequences and their read counts. | | | | | | | |
| --- | --- | --- | --- | --- | --- | --- | --- |
| miRNA | Sequence | 0dpo Good | 0dpo Poor | 14dpo Good | 14dpo Poor | 28dpo Good | 28dpo Poor |
| ssa-let-7a-1 | TGAGGTAGTAGGTTGTATAGTTCAT | 28,2 | 195,1666667 | 39 | 45,66666667 | 24,16666667 | 32,5 |
| ssa-let-7a-2 | TGAGGTAGTAGGTTGTATAGTTG | 2,8 | 2,166666667 | 6,166666667 | 9,5 | 2,5 | 81 |
| ssa-let-7a-3 | TGAGGTAGTAGGTTGTATAGTTT | 4,8 | 8,666666667 | 4,333333333 | 10,33333333 | 3,333333333 | 11,83333333 |
| ssa-let-7a-4 | CTATACAGTCTATTGCCTTCCT | 30,8 | 42,5 | 22 | 25,16666667 | 35 | 41 |
| ssa-let-7b-1 | TGAGGTAGTAGGTTGTGTGGTTTT | 9 | 26,33333333 | 18,16666667 | 14,16666667 | 9,166666667 | 15,5 |
| ssa-let-7c-1 | TGAGGTAGTAGGTTGTATGGTTAA | 12,4 | 15,33333333 | 10,16666667 | 67,83333333 | 5,833333333 | 10,5 |
| ssa-let-7c-2 | CGGTACAACCTTCTAGCTTTCCT | 3 | 3,333333333 | 5,666666667 | 5,166666667 | 2 | 4,833333333 |
| ssa-let-7d-1 | TGAGGTAGTTGGTTGTATGGTTT | 4,6 | 16,66666667 | 10,66666667 | 11,5 | 4,833333333 | 7,666666667 |
| ssa-let-7e-1 | TGAGGTAGTAGATTGAATAGTTG | 6,8 | 91,33333333 | 6,5 | 19,33333333 | 11,66666667 | 14,16666667 |
| ssa-let-7f | TGAGGTAGTAGATTGTATTGTT | 6,6 | 11,83333333 | 10,5 | 13,16666667 | 11,66666667 | 16,33333333 |
| ssa-let-7g-1 | TGAGGTAGTAGTTTGTATAGTTTTAGGA | 14,4 | 58 | 6,833333333 | 12 | 10,16666667 | 11,33333333 |
| ssa-let-7h | TGAGGTAGTAAGTTGTGTTGTTT | 9,4 | 72,16666667 | 14,83333333 | 24,5 | 16,5 | 15,33333333 |
| ssa-let-7i-1 | TGAGGTAGTAGTTTGTGCTGTT | 9 | 13,66666667 | 14,83333333 | 14,66666667 | 5,833333333 | 10,66666667 |
| ssa-let-7i-2 | CTGCGCAAGCTACTGCCTTGCT | 5,4 | 7,666666667 | 7,833333333 | 8,166666667 | 5 | 8,333333333 |
| ssa-let-7j | TGAGGTAGTAGGTTGGATAGTT | 4,8 | 7,166666667 | 4,5 | 6,333333333 | 3 | 2,833333333 |
| ssa-mir-1-1 | TGGAATGTAAAGAAGTATGTAT | 150,4 | 172 | 94 | 181,5 | 56 | 162 |
| ssa-mir-1-2 | TATGACTATATGACAGC | 2 | 1 | 1,166666667 | 1,333333333 | 3 | 1,833333333 |
| ssa-mir-1-3 | ACATACTTCTTTATGCGTCCATA | 7 | 5 | 5,833333333 | 5 | 10 | 5,666666667 |
| ssa-mir-1-4 | ACATACTTCTTTATATGCCCATACG | 14 | 12 | 9 | 15,33333333 | 9,333333333 | 23,33333333 |
| ssa-mir-100a-1 | AACCCGTAGATCCGAACTTGTGCT | 129,4 | 202,1666667 | 75,5 | 165,6666667 | 43,16666667 | 146,1666667 |
| ssa-mir-100a-2 | AACCCGTAGATCCGAACTTGTGTCG | 18,2 | 171,6666667 | 81,16666667 | 47,66666667 | 10,83333333 | 27,5 |
| ssa-mir-101a-1 | GTACAGTACTATGATAACTGAAG | 9 | 20 | 10,33333333 | 12 | 9 | 20 |
| ssa-mir-101b-1 | CAGTTATCATGGTACCGGTGCTAT | 10,4 | 14,83333333 | 8,333333333 | 10,16666667 | 5,5 | 11 |
| ssa-mir-101b-2 | TGTGTGCCTGTCAAG | 1 | 0,333333333 | 3,166666667 | 4,666666667 | 0,333333333 | 1,166666667 |
| ssa-mir-103 | AGCAGCATTGTACAGGGCTATGA | 65,8 | 175,3333333 | 61,5 | 77,66666667 | 20 | 83,5 |
| ssa-mir-106a-1 | TAAAGTGCTTATAGTGCAGGTAAT | 17 | 14,33333333 | 12,16666667 | 20,5 | 9,5 | 29,83333333 |
| ssa-mir-106a-2 | TCTGCCGCAATGTGAGCACTTCT | 1,8 | 3,166666667 | 1 | 2,166666667 | 1,666666667 | 2 |
| ssa-mir-106b | AAAAGTGCTTACAGTGCAGGTA | 10,8 | 11,66666667 | 7,333333333 | 8,166666667 | 5,666666667 | 25,66666667 |
| ssa-mir-107 | AGCTTCTTTACAGTGTTGCCTTGT | 6 | 12,33333333 | 7,5 | 11 | 5,833333333 | 14 |
| ssa-mir-10a-1 | TACCCTGTAGATCCGGATTTGTGT | 64,8 | 104,8333333 | 62 | 100,5 | 23,5 | 249,8333333 |
| ssa-mir-10a-2 | CAAATTCATATCTAAGGGAGT | 0,8 | 1,666666667 | 1,333333333 | 1 | 1 | 1,833333333 |
| ssa-mir-10b-1 | ACCCTGTAGAACCGAATTTGTGTA | 64,8 | 62,16666667 | 34 | 99,83333333 | 14 | 63,33333333 |
| ssa-mir-10b-2 | AGATTCGATTCTAGGG | 5,4 | 5,5 | 7,5 | 5,5 | 2,666666667 | 7,5 |
| ssa-mir-10d-1 | CACCCTGTAGAACCGAATTTGT | 5,2 | 4,5 | 9,666666667 | 7,666666667 | 2,5 | 8,666666667 |
| ssa-mir-122-1 | TGGAGTGTGACAATGGTGTTTGAG | 15,6 | 94,33333333 | 27,33333333 | 33,66666667 | 14,83333333 | 16,66666667 |
| ssa-mir-122-2 | AACGCCATTATCACACTAAATA | 6 | 13,33333333 | 4,333333333 | 6,333333333 | 4 | 5,166666667 |
| ssa-mir-125a-1 | TCCCTGAGACCCTAACTTGTGACG | 115,2 | 526 | 118 | 155,8333333 | 45,33333333 | 144 |
| ssa-mir-125a-2 | TCCCTGAGACCCTAACTTGTGAG | 6,8 | 7,5 | 4,666666667 | 11,83333333 | 5,166666667 | 18 |
| ssa-mir-125b-1 | TCCCTGAGACCCTTAACCTGTGA | 21,6 | 126,8333333 | 33,33333333 | 38,66666667 | 15,16666667 | 30,5 |
| ssa-mir-125b-2 | ACAGGTGAGGTCCTTGGGAAC | 6 | 2,666666667 | 6 | 7,333333333 | 4,833333333 | 6,333333333 |
| ssa-mir-125b-3 | TCCCTGAGACCCTTAACCTGTGAC | 4,8 | 6,166666667 | 10,16666667 | 8,166666667 | 4,166666667 | 3,5 |
| ssa-mir-126-1 | CATTATTACTTTTGGTACGCGCT | 57,6 | 174,1666667 | 50,33333333 | 77,66666667 | 18,5 | 105,8333333 |
| ssa-mir-126-3 | CAAACTCAACTCGGA | 1,6 | 0,333333333 | 0,833333333 | 1,166666667 | 0,333333333 | 1,166666667 |
| ssa-mir-128-1 | CTCACAGTGAACCGGTCTCTTT | 48,6 | 231,5 | 22,16666667 | 37,66666667 | 17,83333333 | 58,5 |
| ssa-mir-129-1 | AAGCCCTTACCCCAAAAAGCAT | 17,8 | 34 | 17,5 | 98,16666667 | 13,5 | 33,33333333 |
| ssa-mir-130a-1 | CTCTTTCCCTGTTGCACTACTATGG | 35282,6 | 49165,16667 | 7307,5 | 11465,66667 | 3902,5 | 3229 |
| ssa-mir-130a-2 | CAGTGCAATAATGAAAGGGCATG | 288,6 | 304,5 | 266,3333333 | 352,3333333 | 121,6666667 | 415,5 |
| ssa-mir-130b-1 | CCCCTTTTGTATTGTACTACTCG | 85 | 95,66666667 | 51,83333333 | 85,66666667 | 56,83333333 | 124,8333333 |
| ssa-mir-130b-2 | TGCCCCTTTTATATTGTCCTACT | 9,4 | 13,5 | 26 | 16,5 | 7,833333333 | 15,66666667 |
| ssa-mir-130d-1 | AGTGCAATATTAAAAGGGCA | 329,2 | 404,8333333 | 235,1666667 | 527 | 212,5 | 466,1666667 |
| ssa-mir-130d-3 | GCCCCTTTTCTATTGCACTACTCG | 9 | 10,33333333 | 8 | 9,666666667 | 3,833333333 | 8 |
| ssa-mir-132-1 | ACCGTGGCTTTAGATTGTTACT | 5 | 13,66666667 | 10 | 8,333333333 | 5,5 | 8,166666667 |
| ssa-mir-1338 | ATCTCAGGTTCGTCAGCCCATG | 7,8 | 14,16666667 | 10,16666667 | 10,33333333 | 6,833333333 | 9,333333333 |
| ssa-mir-133a-1 | AGCTGGTAAAATGGAACCAAATC | 58,6 | 52,16666667 | 54,16666667 | 68,16666667 | 18,83333333 | 64 |
| ssa-mir-133a-2 | CAACTGTTGAATGGATTT | 1 | 0,5 | 0,833333333 | 1,333333333 | 0,833333333 | 0,833333333 |
| ssa-mir-133a-3 | AGCTGGTAAAAAGGAACCAAATG | 2,4 | 3,333333333 | 5,666666667 | 5 | 1,666666667 | 6,333333333 |
| ssa-mir-133b-1 | GCTGGTCAAACGGAACCAAGTC | 3 | 10,16666667 | 5,5 | 10,5 | 2,5 | 4,833333333 |
| ssa-mir-133b-2 | AGGTGTTTCTGTGAGGT | 0,6 | 0,666666667 | 0,833333333 | 1 | 1 | 0,333333333 |
| ssa-mir-135a | TATGGCTTTTTATTCCTATCTGACG | 58,8 | 154,8333333 | 32,83333333 | 82,16666667 | 26,33333333 | 80 |
| ssa-mir-135b-1 | TATGGCTTTCTATTCCTATGTGATG | 23 | 49,33333333 | 19,16666667 | 31 | 11 | 35,16666667 |
| ssa-mir-135b-2 | TATGGCTTTCTATTCCTATGTGATGTT | 4,6 | 6,833333333 | 1,666666667 | 2,5 | 2,166666667 | 2,166666667 |
| ssa-mir-135b-3 | CATAGGGTCCAAAGCCATTG | 1,2 | 3,333333333 | 1,166666667 | 1,333333333 | 1,166666667 | 2,166666667 |
| ssa-mir-135c-1 | TATGGCTTTTTATTCCTACGTGACG | 7,6 | 21,83333333 | 11,16666667 | 19,66666667 | 6,5 | 11 |
| ssa-mir-135c-2 | TATGGCTTTTTATTCCTACGTTAGGG | 1,2 | 1,5 | 0,333333333 | 0,833333333 | 1 | 0,833333333 |
| ssa-mir-137-1 | TTATTGCTTGAGAATACGCGT | 7,8 | 6,166666667 | 6,333333333 | 11 | 8,666666667 | 10 |
| ssa-mir-138-1 | AGCTGGTGTTGTGAATCAGGCCGT | 29,8 | 55,5 | 13,16666667 | 14,66666667 | 16,5 | 32 |
| ssa-mir-139-1 | TCTACAGTGCATGTGTCTCCAGT | 10 | 45 | 7,833333333 | 7 | 3,5 | 9,833333333 |
| ssa-mir-140 | CAGTGGTTTTACCCTATGGTAGTT | 125,6 | 125,5 | 99,16666667 | 147,5 | 55,66666667 | 149,3333333 |
| ssa-mir-142a-1 | TGTAGTGTTTCCTACTTTATGGAA | 7,4 | 9 | 9,5 | 9,166666667 | 6 | 11,16666667 |
| ssa-mir-142a-2 | TAAACTCCTCGCCAC | 1,6 | 3 | 1,333333333 | 2,833333333 | 0,833333333 | 1,833333333 |
| ssa-mir-142b | TCCATAAAGTAGAGAGTACTAC | 3,4 | 5,833333333 | 5,833333333 | 2,5 | 2,833333333 | 3,166666667 |
| ssa-mir-143 | TGAGATGAAGCACTGTAGCTC | 36,2 | 42 | 23,16666667 | 30,66666667 | 31 | 67,33333333 |
| ssa-mir-144 | GGATATCATCATATACTGTAAGTTCG | 16 | 18,66666667 | 13,66666667 | 20,16666667 | 8,5 | 22 |
| ssa-mir-145-1 | GTCCAGTTTTCCCAGGAATCCCTCG | 94,4 | 131,3333333 | 102 | 114,8333333 | 40,16666667 | 145,3333333 |
| ssa-mir-146a-1 | TGAGAACTGAATTCCATAGATGGT | 40,6 | 37,33333333 | 24,33333333 | 29,33333333 | 21,16666667 | 52 |
| ssa-mir-146a-3 | ATCTATGGATTCAGTTC | 1,8 | 2,5 | 2,333333333 | 4,166666667 | 2,166666667 | 2,666666667 |
| ssa-mir-146b | TGAGAACTGAAGTCCA | 0,2 | 1,166666667 | 1 | 0,833333333 | 1,666666667 | 3,5 |
| ssa-mir-146d-1 | TGAGAACTGAATTCCATGGGTTGT | 11,2 | 10,33333333 | 27,83333333 | 8,5 | 7,5 | 11,33333333 |
| ssa-mir-146d-2 | GCCCATGTGGCTCAG | 2,2 | 1,333333333 | 1,166666667 | 3,333333333 | 1 | 2 |
| ssa-mir-148a | AAGTTCTGTGATACACTTCGACT | 64,2 | 79,5 | 49,16666667 | 72,33333333 | 44,33333333 | 114,5 |
| ssa-mir-148b | AAGTTCGGTGATACACTTCGACT | 29 | 27,33333333 | 22,5 | 29,5 | 34,33333333 | 38,33333333 |
| ssa-mir-150 | TCTCCCAATCCTTGTACCAGTGTCT | 9,4 | 24,83333333 | 11 | 15 | 18,5 | 34,83333333 |
| ssa-mir-152 | TCAGTGCATAACAGAACTTTGG | 23,6 | 72 | 16,66666667 | 27,5 | 11,16666667 | 37,83333333 |
| ssa-mir-153a-1 | TTGCATAGTCACAAAAATGATC | 11 | 12,83333333 | 11,5 | 16,33333333 | 6,5 | 16,16666667 |
| ssa-mir-153b | TTGCATAGTCACAAAAGTGATC | 6,2 | 11,66666667 | 9 | 12 | 6,666666667 | 8,333333333 |
| ssa-mir-155-1 | TTAATGCTAATCGTGATAGGGGT | 6,8 | 15,33333333 | 9,166666667 | 14,66666667 | 4,333333333 | 8,333333333 |
| ssa-mir-15a | TAGCAGCACGTCATGGTTTGTAG | 10,2 | 17,5 | 20,66666667 | 23,66666667 | 9 | 17,5 |
| ssa-mir-15b | TAGCAGCACATCATGTTTTGCA | 33,2 | 26,83333333 | 23,66666667 | 38 | 16 | 61,16666667 |
| ssa-mir-15c-1 | TAGCAGCGCATCATGGTTTGAAA | 18,4 | 29,83333333 | 16,16666667 | 34,16666667 | 13,66666667 | 26,83333333 |
| ssa-mir-15d | TAGCAGCACAGAATGGTTTGTCG | 9,4 | 20,5 | 10,33333333 | 14,66666667 | 38,5 | 36,16666667 |
| ssa-mir-15e | AAGCAGCACAGAATGGTTTGT | 1,4 | 9 | 2,5 | 3 | 1,5 | 1,833333333 |
| ssa-mir-16a-1 | TAGCAGCACGTAAATATTGCAGTTA | 26,6 | 123,5 | 31,16666667 | 67 | 42 | 57,66666667 |
| ssa-mir-16b | TAGCAGCACGTAAATATTGGTA | 4,8 | 11,33333333 | 5 | 7,5 | 3 | 5,333333333 |
| ssa-mir-16c | TAGCAGCACGTAAATATTGGCGA | 8,8 | 11,16666667 | 16,83333333 | 16,66666667 | 4,166666667 | 7,5 |
| ssa-mir-17-1 | CAAAGTGCTTACAGTGCAGGTAGTT | 108,4 | 93,16666667 | 100,1666667 | 150,8333333 | 35,83333333 | 106,5 |
| ssa-mir-17-3 | CAAAGTGCTTACAGTGCAGGTAGCG | 1,8 | 6,666666667 | 2,5 | 5,666666667 | 1,666666667 | 3 |
| ssa-mir-1788 | CAGGCAGCTAAAGCAAGTCTC | 4,8 | 6,166666667 | 6 | 19,16666667 | 4,666666667 | 4,666666667 |
| ssa-mir-181a-1 | AACATTCAACGCTGTCGGTGAGTTTA | 159 | 208,3333333 | 78,5 | 159,5 | 90,16666667 | 207 |
| ssa-mir-181a-2 | AACCATCGACCGTTGATTGTACC | 14 | 27,66666667 | 7,166666667 | 6,5 | 4,833333333 | 9 |
| ssa-mir-181a-4 | ACCATCGACCGTTGAGTGTACC | 4,2 | 4,666666667 | 5 | 8,5 | 3 | 6,666666667 |
| ssa-mir-181a-5 | AACATTCAACGCTGTCGGTGAGTTTCG | 3 | 5 | 2,666666667 | 3,333333333 | 3,833333333 | 2,833333333 |
| ssa-mir-181b | AACATTCATTGCTGTCGCTGGGTT | 90,2 | 123,3333333 | 73,16666667 | 135,1666667 | 43 | 131,1666667 |
| ssa-mir-181c | AACATTCATTGCTGTCGGTGGGTTTT | 28,4 | 31,5 | 18,66666667 | 32,16666667 | 10,5 | 42,5 |
| ssa-mir-182 | TTTGGCAATGGTAGAACTCACACCG | 15,4 | 35,83333333 | 13,5 | 14,83333333 | 6,333333333 | 16,33333333 |
| ssa-mir-183-1 | TATGGCACTGGTAGAATTCACTGC | 12,2 | 30,5 | 8 | 13,16666667 | 5 | 12,5 |
| ssa-mir-18a | TAAGGTGCATCTAGTGTAGTTAG | 22,2 | 22 | 16,16666667 | 23,16666667 | 11 | 29,83333333 |
| ssa-mir-18b | TAAGGTGCATCTAGTGCAGATAGCG | 6,8 | 33,5 | 7,166666667 | 5,833333333 | 4,166666667 | 12,83333333 |
| ssa-mir-190a | ATGTTTGATATATTAGGTTA | 5,4 | 9,833333333 | 5,666666667 | 6 | 4,666666667 | 5,833333333 |
| ssa-mir-190b | ACTAAATATCAGACATATTCCTA | 9,8 | 12,16666667 | 13,5 | 9,833333333 | 13,16666667 | 19,66666667 |
| ssa-mir-192a-1 | ATGACCTATGAATTGACAGCCAT | 54 | 58,33333333 | 24,16666667 | 55,66666667 | 18,5 | 56,83333333 |
| ssa-mir-192a-2 | CCTGTCAGTTCTGGAGGC | 1 | 1,333333333 | 0,666666667 | 3,333333333 | 1,333333333 | 1,333333333 |
| ssa-mir-192b | GCGCACACAGGGGTT | 5,2 | 5,833333333 | 24,16666667 | 29,5 | 5,333333333 | 3,666666667 |
| ssa-mir-193 | AACTGGCCCGCAAAGTCCCGCT | 6 | 10,33333333 | 9,333333333 | 10,66666667 | 4,166666667 | 5,666666667 |
| ssa-mir-194a-1 | TGTAACAGCAACTCCATGTGGAAA | 42 | 77,5 | 31 | 45,16666667 | 25,16666667 | 18 |
| ssa-mir-194b | TGTAACAGCATCTCCATATGGAT | 14,6 | 16,16666667 | 9 | 12 | 5,5 | 12 |
| ssa-mir-196a-1 | TAGGTAGTTTCATGTTGTTGGG | 9,6 | 11,5 | 10,83333333 | 17 | 6,166666667 | 16,33333333 |
| ssa-mir-196a-3 | CCGCAACAAGAAACTGCCTTGA | 1,4 | 5,666666667 | 1,666666667 | 4,5 | 2,833333333 | 3,666666667 |
| ssa-mir-196a-4 | CGGCAACAAGAAACT | 0,2 | 0,166666667 | 0,166666667 | 0,333333333 | 0,833333333 | 0,833333333 |
| ssa-mir-196b-1 | TAAGTAGTTTCAAGTTGTTGGG | 10 | 17,16666667 | 11,33333333 | 11,66666667 | 6 | 8,666666667 |
| ssa-mir-199a-1 | CCCAGTGTTCAGACTACCTGTTCCG | 287,8 | 257,3333333 | 186 | 365,3333333 | 83,5 | 355,8333333 |
| ssa-mir-199a-2 | CACAGTAGTCTGCACATTGGTT | 19,2 | 22,5 | 13,5 | 26,83333333 | 9 | 26 |
| ssa-mir-199a-3 | CCCAGTGTTCAGACTACCTGTTCAT | 2,6 | 3,666666667 | 1,666666667 | 1,333333333 | 0,166666667 | 2,666666667 |
| ssa-mir-199a-4 | GAACAGTAGTCTGCACATTGGTT | 18,8 | 14,83333333 | 12,16666667 | 16,5 | 4,833333333 | 17,33333333 |
| ssa-mir-19a-1 | TTGTGCAAATCTATGCAAAACTGA | 90,8 | 107,6666667 | 57,66666667 | 102 | 47,33333333 | 118,6666667 |
| ssa-mir-19a-2 | TGTGCAAATCTATGCAAAACTCT | 1,2 | 0,166666667 | 0,666666667 | 1 | 0,5 | 1,833333333 |
| ssa-mir-19a-3 | CATAGGTGCATTACA | 2,4 | 3 | 4,666666667 | 4,166666667 | 1,333333333 | 1,833333333 |
| ssa-mir-19c-1 | TGTGCAAATCCATGCAAAACTC | 378,4 | 311,6666667 | 288,8333333 | 494,5 | 159,8333333 | 512,3333333 |
| ssa-mir-19c-4 | AGTTTTGCTGGTTTGCTTTCA | 0,2 | 0,166666667 | 0,5 | 0,333333333 | 0,5 | 0,5 |
| ssa-mir-19d | TGTGCAAACCCAAGCAAAACTC | 498,2 | 300,8333333 | 315,6666667 | 296,6666667 | 275,8333333 | 847,6666667 |
| ssa-mir-200a-1 | TAATACTGCCTGGTAATGATGAT | 31,8 | 31,33333333 | 24,16666667 | 48,16666667 | 18,66666667 | 40,83333333 |
| ssa-mir-200a-2 | CATCTTACGAGGCAGCATTGGAC | 7,8 | 9,5 | 6,833333333 | 11 | 5,666666667 | 8,833333333 |
| ssa-mir-200a-3 | CATCTTACGAGGTAGTTTTGG | 0,6 | 2,166666667 | 0,5 | 1,833333333 | 1,666666667 | 1,666666667 |
| ssa-mir-200b-1 | TAACACTGTCTGGTAACGATGTT | 58 | 70 | 34 | 66,83333333 | 22 | 64,66666667 |
| ssa-mir-202 | AAGAGGCATAGGGCATGGG | 101,4 | 242,5 | 68,66666667 | 193,3333333 | 316,6666667 | 172,8333333 |
| ssa-mir-203a-1 | TGTGAAATGTTTAGGACCACTTG | 957 | 1001 | 721,1666667 | 1249,833333 | 366,1666667 | 1319,5 |
| ssa-mir-203a-2 | AGTGGTTCTTAACAGTTCAACAG | 14,2 | 17,66666667 | 9,833333333 | 17,33333333 | 8 | 17,83333333 |
| ssa-mir-203b | AGTGGTTCTTGACAGTTCAACAG | 30 | 76,16666667 | 30,83333333 | 193,8333333 | 19,66666667 | 72 |
| ssa-mir-204-1 | TTCCCTTTGTCATCCTATGCCTGA | 83,4 | 124 | 53,66666667 | 90,5 | 29,16666667 | 94,83333333 |
| ssa-mir-205a-1 | TCCTTCATTCCACCGGATCCTC | 67 | 61,33333333 | 48,66666667 | 83,5 | 26,33333333 | 88,33333333 |
| ssa-mir-205b-1 | TCCTTCATTCCACCGGAGTCTGTT | 50,4 | 56,66666667 | 23,5 | 50,66666667 | 22,16666667 | 49,5 |
| ssa-mir-205b-2 | TTCAGTGGTGTGAAGAGTA | 3,4 | 4 | 4,5 | 6 | 3,166666667 | 7,333333333 |
| ssa-mir-206 | ACATGCTTCCTTATATGCCCATA | 78,2 | 59 | 56,83333333 | 62,5 | 16,66666667 | 51,5 |
| ssa-mir-20a-1 | TAAAGTGCTTATAGTGCAGGTAGT | 33,6 | 210,3333333 | 18 | 35,5 | 13,33333333 | 35,33333333 |
| ssa-mir-20a-2 | TAAAGTGCTTATAGTGCAGGTAGTT | 8 | 7 | 7,666666667 | 4,666666667 | 3,666666667 | 10,16666667 |
| ssa-mir-20a-3 | ACTGCAGTGTGAGCACTTGATGT | 7,4 | 8 | 30,83333333 | 10,5 | 4,833333333 | 215,3333333 |
| ssa-mir-20b | CAAAGTGCTCACAGTGCAGGTAG | 7,8 | 6,5 | 11,5 | 18 | 3,333333333 | 11,83333333 |
| ssa-mir-210-1 | AGCCACTGACTAACGCACATTG | 14,6 | 26,83333333 | 11,5 | 15,5 | 9,5 | 20,5 |
| ssa-mir-212a-1 | ACCTTGGCTATAGACTGCTTACT | 2,8 | 8,833333333 | 3,666666667 | 6,666666667 | 3 | 3,5 |
| ssa-mir-214-1 | TACAGCAGGCACAGACAGGCAGA | 115,2 | 145 | 111,8333333 | 238 | 145,1666667 | 243,6666667 |
| ssa-mir-214-2 | TATCCTGTACAGCAG | 0,6 | 2,333333333 | 2 | 2 | 1 | 0,666666667 |
| ssa-mir-216a | TAATCTCTGCAGGCAACTGTGA | 9 | 9,166666667 | 9,333333333 | 10,16666667 | 5,833333333 | 8 |
| ssa-mir-216b | TAATCTCAGCTGGCAACTGTGAGC | 5 | 12,16666667 | 5,166666667 | 7,166666667 | 3,333333333 | 7,833333333 |
| ssa-mir-218-1 | TTGTGCTTGATCTAACCATGTGT | 71 | 220,1666667 | 67 | 81,16666667 | 23,33333333 | 91,5 |
| ssa-mir-218-2 | TTGTGCTTGATCTAACCAGGTGCCC | 0,6 | 1,333333333 | 1,5 | 2,5 | 1,166666667 | 0,5 |
| ssa-mir-2184 | AACAGTAAGAGTTTATGTGCT | 3,6 | 7,5 | 4,333333333 | 7,5 | 4,166666667 | 8,5 |
| ssa-mir-2187 | TTACAGGCTATGCTA | 16 | 3,666666667 | 2,833333333 | 6 | 2,5 | 5,5 |
| ssa-mir-2188 | AAGGTCCAACCTCACATGTCCTGCG | 101 | 101,8333333 | 80,66666667 | 114,6666667 | 40,16666667 | 122,5 |
| ssa-mir-219a-1 | AGCAAAATCAAACAAAACCCCA | 8,4 | 78,83333333 | 8 | 9,666666667 | 4,833333333 | 6,333333333 |
| ssa-mir-219a-2 | AGCAAAATCAAACGAAACCCCA | 0,2 | 1,166666667 | 1 | 1,166666667 | 0,333333333 | 1,5 |
| ssa-mir-219b-1 | AGAATTGTATCTGGA | 2 | 2,5 | 2,5 | 1,666666667 | 1,5 | 2,166666667 |
| ssa-mir-219c-1 | GGAGTTGTGGATGGACAC | 7,6 | 7,166666667 | 10 | 25 | 4,333333333 | 6,166666667 |
| ssa-mir-219c-2 | GGAGTTGTGGATGGACATCACG | 1,2 | 0,666666667 | 2,5 | 1,833333333 | 0,833333333 | 1,5 |
| ssa-mir-21a-1 | TAGCTTATCAGACTGGTGTTGACTTT | 32,4 | 72,33333333 | 25,33333333 | 39,16666667 | 30 | 51,66666667 |
| ssa-mir-21b | TAGCTTATCAGACTGGTGTTGGCTC | 5,6 | 13,33333333 | 6,666666667 | 5,5 | 8,166666667 | 7,166666667 |
| ssa-mir-221 | ACCTAGCATACAATGTAGATTTC | 10 | 35 | 8,5 | 13,33333333 | 4,5 | 11,83333333 |
| ssa-mir-222a-1 | AGCTACATCTGGCTACTGGGTCTC | 28,4 | 34,16666667 | 22,83333333 | 42,5 | 8,833333333 | 32,16666667 |
| ssa-mir-222b | TGCTCAGTAGTCAGTGTAGATCCCG | 221 | 143,8333333 | 63,66666667 | 84,33333333 | 70,16666667 | 239 |
| ssa-mir-22a | AGTTCTTCACTGGCAAGCTTTAA | 35,6 | 99,16666667 | 45,66666667 | 71 | 48 | 52,16666667 |
| ssa-mir-22b-1 | CGTTCTTCACTAGCTAGCTTTA | 5 | 8,5 | 6,833333333 | 9,666666667 | 4,5 | 5 |
| ssa-mir-23a-1 | AATCACATTGCCAGGGATTTCC | 91 | 125,3333333 | 52 | 90 | 35 | 92 |
| ssa-mir-23a-3 | AATCACATTGCCAGGGATTTCCA | 12,2 | 29,83333333 | 10,16666667 | 20 | 7,833333333 | 19,83333333 |
| ssa-mir-23a-4 | GGTGCACAAATATAAAC | 6 | 7,833333333 | 7,333333333 | 7 | 5,166666667 | 8,166666667 |
| ssa-mir-23b | ATCACATTGCCAGGGATTACCACT | 24 | 47,33333333 | 16,33333333 | 41,16666667 | 11,5 | 41,16666667 |
| ssa-mir-24a-1 | TGGCTCAGTTCAGCAGGAAC | 17,2 | 18,83333333 | 22,66666667 | 24,5 | 6,333333333 | 21,5 |
| ssa-mir-24a-3 | TGCCTACTGAGCTGATAACAGT | 2,6 | 2,333333333 | 4,5 | 4,833333333 | 4,166666667 | 4,166666667 |
| ssa-mir-24a-4 | TGCCTGCTGTGCTGATAATCAGT | 4 | 5,666666667 | 5,5 | 1,5 | 0,833333333 | 3,833333333 |
| ssa-mir-24b | TGGCTCAGTTCAGCAGAAACCG | 5,4 | 4 | 2,333333333 | 7,333333333 | 2,333333333 | 6,833333333 |
| ssa-mir-25-1 | CATTGCACTTGTCTCGGTCTGA | 35,2 | 46 | 20,16666667 | 45,66666667 | 9,333333333 | 48,83333333 |
| ssa-mir-25-3 | AGCCATTGCAGACGG | 2 | 4,833333333 | 4,166666667 | 2,833333333 | 2,333333333 | 2,333333333 |
| ssa-mir-26a-1 | TCAAGTAATCCAGGATAGGCTCGTTCC | 81,2 | 144,8333333 | 49,66666667 | 88,16666667 | 17,16666667 | 88 |
| ssa-mir-26a-3 | CCTGTTCTTGATTACTTGTTA | 2 | 2,5 | 14,16666667 | 5,5 | 4,166666667 | 5,666666667 |
| ssa-mir-26a-4 | CGGCCTATTTTTGATTACTTGT | 1,6 | 5,333333333 | 1,666666667 | 2 | 0,833333333 | 2,166666667 |
| ssa-mir-26a-5 | TTCAAGTAATCCAGGATAGGCTTCT | 7,8 | 13,5 | 7,333333333 | 6,5 | 4,166666667 | 13,5 |
| ssa-mir-26a-6 | TTCAAGTAATCCAGGATAGGCTGCG | 5,2 | 9,666666667 | 6,166666667 | 6 | 3,833333333 | 10,33333333 |
| ssa-mir-26b | TTCAAGTAATCCAGGATAGGTTCG | 4 | 8,5 | 4,5 | 7,833333333 | 5 | 8,666666667 |
| ssa-mir-26d | TTCAAGTAATCTAGGATAGGCTT | 0,4 | 2,666666667 | 1,833333333 | 2,666666667 | 1,666666667 | 1,833333333 |
| ssa-mir-27a-1 | TTCACAGTGGCTAAGTTCCGCA | 24,8 | 68,5 | 12,5 | 19,5 | 10,83333333 | 20 |
| ssa-mir-27b-1 | AGAGCTTAGCTGATTGGTGAACA | 7,4 | 37,16666667 | 9,5 | 11,83333333 | 5,166666667 | 8,166666667 |
| ssa-mir-27c-1 | TTCACAGTGGCTAAGTTCAGT | 7,4 | 7,333333333 | 4,5 | 7,166666667 | 3,5 | 4,5 |
| ssa-mir-27d | TTCACAGTGGTTAAGTTC | 4,6 | 7,666666667 | 8,833333333 | 8,333333333 | 3,833333333 | 6 |
| ssa-mir-29a | CTAGCACCATTTGAAATCAGT | 9,4 | 19,16666667 | 8,833333333 | 21,5 | 8,166666667 | 16,83333333 |
| ssa-mir-29b-1 | CTAGCACCATCTGAAATCGGTTA | 20,2 | 68,16666667 | 42 | 15 | 7,666666667 | 25 |
| ssa-mir-29b-2 | ACTGATTTCCTCTGGTGTTTAG | 0,6 | 0,833333333 | 0,5 | 1,666666667 | 0,833333333 | 1,333333333 |
| ssa-mir-29c | CTAGCACCATTTTAAATCAGTTA | 2 | 5,166666667 | 2,666666667 | 3 | 2,833333333 | 3,833333333 |
| ssa-mir-301a-1 | CAGTGCAATAGTATTGTCATAGC | 325,2 | 307,6666667 | 249,8333333 | 390,8333333 | 116,8333333 | 509,8333333 |
| ssa-mir-301b | CAGTGCAACAGTATTGTCATGGC | 37,8 | 34 | 23,16666667 | 44,83333333 | 12,5 | 46,66666667 |
| ssa-mir-301c | CAGTGCAATAGTATTGTCATGGC | 4,8 | 4,333333333 | 6 | 16,83333333 | 4,5 | 16,66666667 |
| ssa-mir-301d | CAGTGCAATAGTATTGTCAAAGC | 8,8 | 14,5 | 6,833333333 | 15,83333333 | 6,166666667 | 22,16666667 |
| ssa-mir-30a-1 | TGTAAACATCCTACACTCTCAGCCG | 280,2 | 170,5 | 92,5 | 102,5 | 93,83333333 | 320,5 |
| ssa-mir-30a-3 | TGTAAACATCCTACACTCTCAGCTTT | 18,6 | 15,33333333 | 19 | 17,33333333 | 6,166666667 | 19 |
| ssa-mir-30b | TGTAAACATCCCCGACTGGAAGCTC | 26,2 | 46,16666667 | 25,16666667 | 38,33333333 | 12,5 | 39,66666667 |
| ssa-mir-30c-1 | TGTAAACATCCTTGACTGGAAGCTC | 26,4 | 40,33333333 | 22,16666667 | 39,33333333 | 11,16666667 | 35 |
| ssa-mir-30d-1 | CTTTCAGTCGGATGTTTGC | 3,2 | 7,5 | 5,166666667 | 7,166666667 | 2,833333333 | 10,16666667 |
| ssa-mir-30d-2 | CTTTCAGTCGGATGTTTGCAGCT | 1 | 0,666666667 | 0,666666667 | 0,5 | 0,333333333 | 1,333333333 |
| ssa-mir-30e-1 | TGTAAACATCCTACACTCAGCTGCG | 12 | 62,83333333 | 9,833333333 | 27,33333333 | 6,5 | 30,66666667 |
| ssa-mir-30e-3 | TGTAAACATCCTACACTCAGCTATA | 7,4 | 4,333333333 | 10,16666667 | 33,33333333 | 3,666666667 | 8,5 |
| ssa-mir-338a-1 | TCCAGCATCAGTGATTTTGTTG | 18,8 | 80,16666667 | 21,33333333 | 29,33333333 | 11,16666667 | 23,16666667 |
| ssa-mir-338a-3 | AACAATATCCTGGTGCTGCCT | 6 | 13 | 18,16666667 | 55,5 | 8,166666667 | 6 |
| ssa-mir-338a-4 | AACAACATCCTGGTG | 2 | 2,166666667 | 3,333333333 | 1,833333333 | 1,5 | 2,833333333 |
| ssa-mir-33a-1 | GTGCATTGTAGTTGCATTGCA | 5 | 6,333333333 | 5,166666667 | 4,5 | 2,166666667 | 14,5 |
| ssa-mir-33b | CAATGTTCCTGCAGTGCAAG | 2,2 | 3,833333333 | 2,666666667 | 2,666666667 | 1 | 2,666666667 |
| ssa-mir-365-1 | TAATGCCCCTAAAAATCCTTAT | 5,8 | 17,83333333 | 8,833333333 | 16,83333333 | 10,16666667 | 16,83333333 |
| ssa-mir-365-2 | AGGGGCTTTAGGGGGC | 2,8 | 4,166666667 | 3,833333333 | 5,166666667 | 4,833333333 | 7 |
| ssa-mir-375-1 | ACGTTGAGCCACACGCACAATAC | 8,2 | 40,83333333 | 40,83333333 | 29,66666667 | 4,666666667 | 10,83333333 |
| ssa-mir-375-3 | ACGTTGAGCCATACGCACAATAC | 0,4 | 3,833333333 | 1 | 1,666666667 | 0,666666667 | 1,833333333 |
| ssa-mir-429 | TAATACTGTCTGGTAATGCCG | 6,2 | 10,16666667 | 4,166666667 | 8,5 | 3,666666667 | 9 |
| ssa-mir-430a | TACCCGAACACTAGCATTGACT | 7,6 | 12,5 | 11,5 | 13,33333333 | 5,5 | 9,833333333 |
| ssa-mir-430b | AAAGTGCTATTAAGTTGGAGTAT | 8,6 | 10,33333333 | 13,16666667 | 12,83333333 | 8,5 | 18,66666667 |
| ssa-mir-430c | AACCCTAACAAAAGCATTGACTCG | 38,2 | 38 | 24,83333333 | 46,33333333 | 10,16666667 | 50,66666667 |
| ssa-mir-449a | GGCAGTGTAACGTTAGCTGAC | 1,2 | 6,833333333 | 3,666666667 | 4,666666667 | 2 | 4,333333333 |
| ssa-mir-449b | AGGCAGTGTCTTGTTAGCTGAT | 6,8 | 12,5 | 4 | 8,833333333 | 5,333333333 | 12,5 |
| ssa-mir-454 | ACCCTATCAATATTGCCTCTGCT | 9,8 | 10,66666667 | 8,666666667 | 16,5 | 6,833333333 | 12,66666667 |
| ssa-mir-455 | AGCACCATGCAGTCCATGGGCATATACA | 30,6 | 38,83333333 | 21,33333333 | 26 | 19,16666667 | 41 |
| ssa-mir-456-1 | CAGGCTGGTTAGATGGTTGTCT | 16,2 | 28,83333333 | 10,66666667 | 24,83333333 | 35,16666667 | 23,16666667 |
| ssa-mir-458 | ATAGCTCTTTAAATGGTACTGC | 11,4 | 10,33333333 | 7,666666667 | 13,16666667 | 5,5 | 6,833333333 |
| ssa-mir-459 | TCAGTAACAAGGATTCATC | 2,2 | 7,666666667 | 19,33333333 | 13,5 | 3 | 5,166666667 |
| ssa-mir-460-1 | CCTGCATTGTACACACTGTGCGTA | 14,8 | 18,33333333 | 18 | 27,83333333 | 6,166666667 | 19,5 |
| ssa-mir-462a | TAACGGAACCCATAATGCAGCTGCG | 4,4 | 25,5 | 8,333333333 | 21,66666667 | 5,333333333 | 7,5 |
| ssa-mir-462b | TAACGGAACCCATAAAGCAGCTGCG | 1,6 | 3,166666667 | 2,833333333 | 4,166666667 | 4,5 | 2,166666667 |
| ssa-mir-489-1 | TGACATCATATGTACGGCTGCT | 2,4 | 20 | 3,5 | 4,333333333 | 1,5 | 4,666666667 |
| ssa-mir-499a | AACATCACTTTAAGTCTCTGCT | 4,2 | 9,166666667 | 6 | 4,833333333 | 6 | 6,333333333 |
| ssa-mir-499b-1 | TTAAGACTTGCAGTGATGTTTAG | 3,4 | 4 | 2,333333333 | 9,166666667 | 1,333333333 | 5,666666667 |
| ssa-mir-551 | GCGACCCATCCTTGGATTCT | 15,6 | 11,5 | 8,833333333 | 17,83333333 | 6,833333333 | 15,16666667 |
| ssa-mir-7132a | GACTTGGTCAAAGCTCCTGAGT | 5,2 | 7,833333333 | 7 | 10,83333333 | 7,166666667 | 10,66666667 |
| ssa-mir-7132b | GACTTGGTCAAAGCTCCTCAGCATAT | 3,8 | 3,666666667 | 4,666666667 | 8,333333333 | 3,666666667 | 30,66666667 |
| ssa-mir-722-1 | CCCAGTAAAGGTGTT | 4 | 7,5 | 11,33333333 | 14,66666667 | 3,666666667 | 8,666666667 |
| ssa-mir-723-1 | GGCAGCTTTGAATGATGTTAC | 5,6 | 11 | 6 | 8,666666667 | 6,5 | 8,833333333 |
| ssa-mir-724-1 | TTAAAGGGAATTTGCGACTGCT | 23,8 | 39,66666667 | 22,33333333 | 24 | 22,83333333 | 34,5 |
| ssa-mir-725-1 | AGCTAGAAACCTTGCCTGAGATT | 4 | 5,666666667 | 9 | 9,666666667 | 4,5 | 9,5 |
| ssa-mir-727-1 | AGTCTTCAATTCCTCCCAGCCCGTG | 5 | 12,16666667 | 7,666666667 | 8 | 3,333333333 | 6,833333333 |
| ssa-mir-730a-1 | TCCTCATTGTGCATGCTGTGTGT | 7,4 | 7,666666667 | 7,166666667 | 5 | 3 | 7,166666667 |
| ssa-mir-731 | AATGACACGTTTTCTCCCGGATTGCCG | 5 | 6,833333333 | 8,333333333 | 13,33333333 | 4,5 | 6,333333333 |
| ssa-mir-734 | GAACTATTCTGCAACATTTGTCG | 7 | 8,666666667 | 5,333333333 | 12 | 7,833333333 | 13,33333333 |
| ssa-mir-737 | GTTTTTTTAGGTTTAGACTTTT | 2,8 | 5 | 5,833333333 | 5,166666667 | 2,166666667 | 5,333333333 |
| ssa-mir-7552a-1 | TCCCTTAATTGTTTAG | 5,6 | 7,333333333 | 8,833333333 | 11,66666667 | 3,666666667 | 10,33333333 |
| ssa-mir-7552b | CTACAATTAAAGGATATTTCT | 2,2 | 4,333333333 | 4,333333333 | 5,166666667 | 2,666666667 | 5,166666667 |
| ssa-mir-7a-1 | TGGAAGACTAGTGATTTTGTTGTT | 11,6 | 370,8333333 | 10,33333333 | 12,16666667 | 3,166666667 | 10,66666667 |
| ssa-mir-7a-2 | TGGAAGACTAGTGATTTTGTTGTGT | 2,6 | 3,5 | 2,666666667 | 8 | 1,333333333 | 2,833333333 |
| ssa-mir-7a-3 | CAACAAATCACAGTCTGCCAAT | 3,8 | 4,333333333 | 2,666666667 | 4,833333333 | 3 | 4,5 |
| ssa-mir-7a-4 | CAACAAGTCACAGTCTACCTC | 2,8 | 6 | 2,5 | 4,333333333 | 1,833333333 | 2 |
| ssa-mir-7a-5 | CAACAAGTCATAGTCTACCTCA | 1 | 2,666666667 | 2,5 | 1,333333333 | 1 | 2,166666667 |
| ssa-mir-8156 | GTCCTGACTGTCCTGAC | 2,8 | 4,166666667 | 5,166666667 | 3,333333333 | 2,666666667 | 2,833333333 |
| ssa-mir-8157 | TGCACTGTACTCTGG | 2 | 3,333333333 | 2,5 | 3,666666667 | 1,166666667 | 2,833333333 |
| ssa-mir-8158 | TTTGTACGTGTGAAACTTCTTCC | 1,2 | 4,166666667 | 2,666666667 | 3,833333333 | 3,5 | 5,666666667 |
| ssa-mir-8159 | TCAGTAACTGGAATCTGTCCCTGC | 9,6 | 7,333333333 | 7 | 8,666666667 | 4,333333333 | 6,5 |
| ssa-mir-8160 | AGAATAATGCCAGCAGTCGGCC | 639,2 | 404 | 237,3333333 | 671,5 | 490,1666667 | 1027,833333 |
| ssa-mir-8162 | TCACAACGGATCTGGG | 2,8 | 2,666666667 | 3,833333333 | 3,666666667 | 3,166666667 | 1,333333333 |
| ssa-mir-8164 | CAGAGGTATTGTAATATCGTGA | 6,6 | 8,5 | 7 | 9 | 7,833333333 | 14 |
| ssa-mir-92a-1 | TATTGCACTTGTCCCGGCCTGA | 56,6 | 104 | 59,66666667 | 93,66666667 | 29,66666667 | 74,83333333 |
| ssa-mir-92a-3 | AGGCTGGGAGGGGTG | 4,4 | 4,666666667 | 4,666666667 | 6,166666667 | 1,666666667 | 7,833333333 |
| ssa-mir-92b | TATTGCACTCGTCCCGGCCTCC | 10,8 | 15 | 7,5 | 16 | 5,166666667 | 12,5 |
| ssa-mir-93a-1 | AAAAGTGCTGTTTGTGCAGGTAG | 10,8 | 13 | 14,5 | 11,83333333 | 5 | 11,83333333 |
| ssa-mir-93a-2 | ACTGCAAAACCAGCACTTCCCGA | 3 | 5,666666667 | 0,833333333 | 8,833333333 | 2,666666667 | 3,333333333 |
| ssa-mir-96-1 | TTTGGCACTAGCACATTTTTGCTTA | 6,6 | 9,166666667 | 4,666666667 | 9,333333333 | 7,333333333 | 7 |
| ssa-mir-99-1 | AACCCGTAGATCCGATCTTGTGA | 101,2 | 170,1666667 | 156,8333333 | 99 | 46,16666667 | 302,3333333 |
| ssa-mir-9a-1 | TCTTTGGTTATCTAGCTGTATGAT | 22,6 | 111,8333333 | 21 | 50,5 | 16,83333333 | 46,5 |
| ssa-mir-9a-2 | ATAAAGCTAGATAACCGAAAGTC | 15 | 26,16666667 | 11,5 | 17,5 | 7 | 21,5 |
| ssa-mir-9a-3 | TAAAGCTAGAGAACCGAAAGTA | 3 | 15,16666667 | 2,5 | 6,5 | 2 | 1,166666667 |
| ssa-mir-9a-4 | CTTCTTAAAGCTAGA | 2,2 | 1,5 | 1,833333333 | 4,666666667 | 3 | 3,333333333 |
| ssa-mir-9a-6 | ATAAAGCTAGCTAACCGAATGT | 5,6 | 6,333333333 | 7,5 | 6,166666667 | 5 | 8,666666667 |
| ssa-mir-9a-7 | ATAAAGCTAGCTAACCGAAAGTC | 0 | 0,333333333 | 0,666666667 | 0,5 | 0,166666667 | 0,5 |
| ssa-mir-9b | TCTTTGGTTATCTAGCTGAATC | 0,2 | 0,5 | 1,166666667 | 0,666666667 | 0,333333333 | 2,166666667 |
