# Supplementary material for: Postovulatory maternal transcriptome in Atlantic salmon and its relation to developmental potential of embryos
Source: BMC Genomics. 2019 Apr 24;20:315. doi: 10.1186/s12864-019-5667-4 (PMC6480738; doi:10.1186/s12864-019-5667-4)
Supplement: Supplementary file 12 — Primers used for RT-qPCR. Gene name and symbol with forward and reverse primer sequences (5′ -3′) are given. (DOCX 19 kb) [file 12864_2019_5667_MOESM12_ESM.docx]

Additional file 11. Primers used for RT-qPCR.

| Gene name | Gene symbol | Forward primer (5’ – 3’) | Reverse primer (5’ – 3’) |
| --- | --- | --- | --- |
| beta actin1 | *actin1* | GGCATCTGACCGGAGATAAA | GGCGATTTCATCTTCCATTT |
| borealin-like transcript variant X2 | *borealin2* | TCCCTGTCTGGATTGTCTCC | TATCATTGCGCACTCAGAGG |
| cathepsin Z-like | *ctsz* | GTGAGCATCACGAGGAACCA | TGATGTTGATACGGTCGGCA |
| F-box only protein 6 like | *fbox6* | TGTCGACTTGTGTGTCGTGA | GCCAGCCCTTGAATTTATCA |
| hepcidin-1 | *hepc1* | TCTGCCGATGCATTTCAGGT | TGGCTTTAGTGCTGGCAGG |
| interferon regulatory factor 7 | *irf7* | CGTCAAGGTGGTTCCCCTG | AGATAGGGGCTGGTGATGTG |
| intracellular hyaluronan-binding protein 4 | *habp4* | CTGAGAAGGCCCGATTCCAG | TCCTGCTCCTCTTGGGAAGT |
| NADH dehydrogenase [ubiquinone] 1 alpha subcomplex subunit 8-like | *ndufa8* | GGGAGAACTGTCCAAGGTGAC | CCCGGGAGTACTGACTACCT |
| nucleoplasmin ATPase-like | *npmATPase* | GTCGGACGATGAGAACCCTC | CAGCAGCACCTGGGATTACT |
| phospholipid hydroperoxide glutathione peroxidase mitochondrial | *phgpx* | TGGATGACCCCATTGTGATCG | CCAAAGGAGAGGTCAGCCAG |
| transcription factor IIIB 90 kDa subunit-like | *tfIIIb* | TGTAAGAACTGTGGGGGCAC | TCCAGAGGCATCTCCAGCTA |
| translocase of inner mitochondrial membrane 8 homolog A | timm8a | CAGACCCTCAGCTTCAGCAA | TCCATGCATTTCTCCCAACAC |
| tripartite motif-containing protein 47-like | *trim47* | ATGGGGAAGAGGAAGAAGGA | AGGGGCAACTTCACCCTACT |
| zinc finger protein 628-like | *znf628* | CCGGAAAAGGACATCTACGA | TACAGTGGTCTCGTCCGTCA |
